# A Mendelian Randomization Analysis of 55 Genetically Predicted Metabolic Traits with Breast Cancer Survival Outcomes in the Pathways Study

**DOI:** 10.1158/2767-9764.CRC-23-0047

**Published:** 2023-06-22

**Authors:** Peter N. Fiorica, Haiyang Sheng, Qianqian Zhu, Janise M. Roh, Cecile A. Laurent, Isaac J. Ergas, Jennifer Delmerico, Marilyn L. Kwan, Lawrence H. Kushi, Christine B. Ambrosone, Song Yao

**Affiliations:** 1 Jacobs School of Medicine and Biomedical Sciences, University at Buffalo, Buffalo, New York.; 2 Department of Cancer Prevention and Control, Roswell Park Comprehensive Cancer Center, Buffalo, New York.; 3 Department of Biostatistics and Bioinformatics, Roswell Park Comprehensive Cancer Center, Buffalo, New York.; 4 Division of Research, Kaiser Permanente Northern California, Oakland, California.

## Abstract

Previous studies suggest associations of metabolic syndromes with breast cancer prognosis, yet the evidence is mixed. In recent years, the maturation of genome-wide association study findings has led to the development of polygenic scores (PGS) for many common traits, making it feasible to use Mendelian randomization to examine associations between metabolic traits and breast cancer outcomes. In the Pathways Study of 3,902 patients and a median follow-up time of 10.5 years, we adapted a Mendelian randomization approach to calculate PGS for 55 metabolic traits and tested their associations with seven survival outcomes. Multivariable Cox proportional hazards models were used to derive HRs and 95% confidence intervals (CI) with adjustment for covariates. The highest tertile (T3) of PGS for cardiovascular disease was associated with shorter overall survival (HR = 1.34, 95% CI = 1.11–1.61) and second primary cancer–free survival (HR = 1.31, 95% CI = 1.12–1.53). PGS for hypertension (T3) was associated with shorter overall survival (HR = 1.20, 95% CI = 1.00–1.43), second primary cancer–free survival (HR = 1.24, 95% CI = 1.06–1.45), invasive disease-free survival (HR = 1.18, 95% CI = 1.01–1.38), and disease-free survival (HR = 1.21, 95% CI = 1.04–1.39). PGS for serum cystatin C levels (T3) was associated with longer disease-free survival (HR = 0.82, 95% CI = 0.71–0.95), breast event-free survival (HR = 0.74, 95% CI = 0.61–0.91), and breast cancer–specific survival (HR = 0.72, 95% CI = 0.54–0.95). The above associations were significant at a nominal *P* < 0.05 level but not after correcting for multiple testing (Bonferroni *P* < 0.0009). Our analyses revealed notable associations of PGS for cardiovascular disease, hypertension, and cystatin C levels with breast cancer survival outcomes. These findings implicate metabolic traits in breast cancer prognosis.

**Significance:**

To our knowledge, this is the largest study of PGS for metabolic traits with breast cancer prognosis. The findings revealed significant associations of PGS for cardiovascular disease, hypertension, and cystatin C levels with several breast cancer survival outcomes. These findings implicate an underappreciated role of metabolic traits in breast cancer prognosis that would warrant further exploration.

## Introduction

Breast cancer is the most common malignancy in women. In 2022, an estimated 287,000 women in the United States will be diagnosed with breast cancer, and an estimated 43,000 will die from the disease (1). Breast cancer has a well-established causal genetic component, including a few rare but high penetrance genetic variants and hundreds of common but low penetrance variants that have been identified through genome-wide association studies (GWAS; refs. 2–4). In comparison, far fewer SNPs have been identified in association with breast cancer survival outcomes through GWAS (5–11). As a result, we still know little about the role of germline genetic underpinnings for breast cancer prognosis.

Although identification of *bona fide* genetic variants associated with breast cancer survival awaits future large studies with well-annotated outcome data, an alternative approach is Mendelian randomization. This method relies on genetic instruments defined by individual variants, or more recently polygenic scores (PGS), that mathematically aggregate the effects of tens to millions of SNPs, instead of the traits themselves, in tests for associations with outcomes of interest. This is attractive because genetic scores are fixed, may represent the lifetime baseline levels of the traits measured, and may not be subject to reverse causality (12, 13). The maturation of the GWAS literature has made it possible for studies to leverage the findings from well-powered GWAS of many common traits and conduct Mendelian randomization analysis of those traits in association with outcomes of interest. PGS is a score that represents the totality of an individual's genetic determinants of a trait and can be computed on the basis of an algorithm developed in well-powered GWAS for the trait. With the continuing maturation of the GWAS literature and development of newer statistics for calculating PGS, it is possible that in a near future, these genetic scores can have clinical values for risk stratification and survival prognostication purpose. Currently, no germline genetic factors have been used clinically for breast cancer prognosis.

Recently, metabolic syndrome, a group of conditions including obesity, hypertension, hyperglycemia, and dyslipidemia, has been investigated in relation to breast cancer survival. These conditions are highly prevalent in the United States and affect a large proportion of women, including those diagnosed with breast cancer. In an earlier study, low levels of high-density lipoprotein and high triglycerides were found to be associated with increased risk of new breast cancer events (locoregional recurrences, distant metastasis, and new primary breast cancer diagnosis; ref. 14). A more recent meta-analysis reported that metabolic syndrome was associated with all-cause mortality but not breast cancer–specific mortality (15). However, because these metabolic conditions vary across the lifetime and may change through the course of cancer development and treatment, the causal relationship between these conditions and poor breast cancer prognosis has yet to be established.

In this study, we adapted the Mendelian randomization approach and investigated the associations of PGS for 55 metabolic phenotypes where results from large GWAS were available with survival outcomes in 3,902 women diagnosed with breast cancer from the Pathways Study, one of the largest prospective studies of breast cancer survivors.

## Materials and Methods

### Study Population

The Pathway Study is a large prospective cohort of 4,505 breast cancer survivors enrolled shortly after cancer diagnosis between 2006 and 2013 at Kaiser Permanente Northern California (KPNC) with ongoing follow-up. The study design, data collection, and patient follow-up are described in detail elsewhere (16). Participants in the Pathways Study provided extensive information on demographics and epidemiologic risk factors through questionnaires administered at baseline and follow-up assessments. Data on cancer histopathologic features, treatments received, and various survival outcomes were obtained and integrated from established electronic clinical databases. In accordance with the Declaration of Helsinki for ethical guidelines, Institutional Review Boards at Roswell Park Comprehensive Cancer Center and KPNC approved this study, and all participants provided informed consent.

### Genotype Data and Calculation of PGS

Of the 4,505 individuals in the Pathways Study, genotypes and survival phenotypes from 3,902 individuals were available for our analyses after quality control. Peripheral blood and/or saliva samples were collected at the time of enrollment and used as sources of germline DNA. Genotyping was performed for all participants with DNA samples available by the Center for Inherited Disease Research (CIDR), using the Illumina Multi-Ethnic Genotyping Array with custom content from the BioVU breast cancer SNP subset. Standard quality control (QC) was performed after genotyping, and imputation was performed using the University of Michigan Imputation Server and the Haplotype Reference Consortium reference panel (17). The genotyping assays, QC, and imputation are described in detail elsewhere (6). To calculate PGS for metabolic traits, variants and associated weights were obtained from the PGS Catalog (18). We define a metabolic trait as a biomarker that is clinically used for testing and monitoring metabolic syndromes and disease conditions that may arise as sequalae of the metabolic derangement. A total of 55 PGS were included in the analysis. Details of those PGS are provided in Supplementary Table S1. PGS calculation was done using all variants that could be matched in the genotyped and imputed data. Because data QC and imputation were conducted separately in each of the four major racial and ethnic groups (White, Black, Asian, and Hispanic) as defined by self-report, PGSs were also calculated separately within each group.

### Statistical Analysis

Descriptive characteristics of cohort members are summarized using standard univariate statistics. Each PGS was categorized into tertiles within each of the four racial and ethnic groups before being combined for analysis. Seven survival outcomes were analyzed, including overall survival (event was death due to any cause), breast cancer–specific survival (event was death attributed to breast cancer), recurrence-free survival (event was recurrence or death due to any cause), second primary cancer–free survival (event was secondary primary cancer or death due to any cause), disease-free survival (event was recurrence, second primary invasive or *in situ* breast cancer, or death due to any cause), invasive disease-free survival (event was recurrence, second primary invasive breast cancer, or death due to any cause), and breast cancer event-free survival (event was recurrence, second primary invasive or *in situ* breast cancer, or death due to breast cancer). Deaths are obtained through KPNC mortality databases, the State of California, and the U.S. Social Security Administration. Cause of death is obtained from the National Death Index (NDI) and electronic health record (EHR). Recurrences are obtained by yearly follow-up via phone interview, or linkage to the EHR or KPNC Cancer Registry, and then verified with medical record review. The current dropout rate for Pathways Study is 13%, which counts patients who have dropped out of the Kaiser Permanente health care plan. For those patients, passive follow-up on death continues through the NDI. Time to event was calculated from the date of diagnosis to the time of event of interest using patient age as the time scale. Patients without an event of interest were censored at the time of disenrolling from the Kaiser Permanente health plan or the date of the last update of survival outcomes (December 31, 2019).

The estimated HR and 95% confidence interval (CI) were obtained from Cox proportional hazards regression models, with the first tertile (T1) used as the reference group while adjusting for age at diagnosis, race and ethnicity group, tumor stage, tumor grade, IHC subtype, surgery, radiotherapy, chemotherapy, and endocrine therapy. The proportional hazards assumption of each covariate was checked using Schoenfeld residuals, and when the assumption was violated, an interaction term with survival time (time-varying coefficient) was added to the regression model. Time-varying covariates for each test are listed in Supplementary Table S2. The significance of the association between a PGS and a survival outcome was examined by the likelihood ratio test comparing the nested models before and after removing the PGS tertiles from the model. For outcomes other than those that included overall survival, competing risk models were also tested by treating death due to cardiovascular causes as a competing risk. We chose cardiovascular disease (CVD) causes of death as a competing risk because CVD is among the top noncancer causes of death among patients with breast cancer. CVD-related causes of death included myocardial infarction, stroke, and other deaths attributable to complications of the cardiovascular system. In addition, CVD is related to many of the metabolic traits we consider in our tests. We further stratified our analysis by tumor estrogen receptor (ER) subtype to detect associations potentially specific to ER-positive (ER^+^) and ER-negative (ER^−^) tumors. A nominal *P* value ≤0.05 was used as the cutoff for significance. Multiple testing correction for 55 PGS was conducted using the Bonferroni approach (*P* < 0.0009). All statistical analyses were performed in R, version 4.2.0.

### Data Availability Statement

Genotype data from the Pathways Study have been deposited in dbGaP along with survival data and other key covariate data (study accession: phs001534.v1.p1). Data used for this analysis are available from the corresponding author upon reasonable request.

## Results

### Patient Characteristics and Survival Outcomes

Descriptive characteristics of the 3,902 patients with breast cancer are summarized in Table 1. The median age at diagnosis was 59.8 (range: 23.6–94.8) years, with 71% diagnosed postmenopause. Most (69.1%) individuals self-identified as White, 12.2% as Asian, 7.6% as Black, and 11.2% as Hispanic. The median body mass index (BMI) was 27.4 (range: 14.1–67.6) kg/m^2^, with approximately each third of the patient population being normal weight, overweight, and obese. Most women had ER^+^ tumors (83.6%), were diagnosed at early stages (89.1% stages I and II) with grade I (28.3%) or grade II (45.6%) disease. The majority (60.1%) of women received lumpectomy rather than mastectomy (36.9%) and received some type of adjuvant therapy (44.4% radiotherapy, 47.0% chemotherapy, and 74.9% hormonal therapy). The median follow-up time of the Pathways Study at the time of analysis was 10.5 (range: 0.2–14.2) years. Survival events are summarized by Table 2.

**TABLE 1:** Baseline characteristics of patients with breast cancer in the Pathways Study (*n* = 3,902)

	*n* (%)
**Age at breast cancer diagnosis, years, *n* (%)**	
<50	837 (21.5%)
50–59	1,137 (29.1%)
60–69	1,143 (29.3%)
≥70	785 (20.1%)
Age at breast cancer diagnosis, median (range), years	59.8 (23.6, 94.8)
**Menopausal status at breast cancer diagnosis**	
Premenopausal	1,144 (29.3%)
Postmenopausal	2,758 (70.7%)
**Race/ethnicity**	
White	2,696 (69.1%)
Black	296 (7.6%)
Asian	474 (12.2%)
Hispanic	436 (11.2%)
**Body mass index at breast cancer diagnosis, kg/m^2^**	
<25 kg/m^2^	1,304 (33.4%)
25–29.9 kg/m^2^	1,221 (31.3%)
≥30 kg/m^2^	1,377 (35.3%)
Body mass index at breast cancer diagnosis, median (range), kg/m^2^	27.4 (14.1, 67.6)
**AJCC stage**	
I	2,138 (54.8%)
II	1,338 (34.3%)
III	370 (9.5%)
IV	56 (1.4%)
**Tumor grade**	
Grade 1	1,038 (28.3%)
Grade 2	1,669 (45.6%)
Grade 3	957 (26.1%)
**Tumor ER status**	
Positive	3,260 (83.6%)
Negative	640 (16.4%)
**Tumor PR status**	
Positive	2,495 (64.0%)
Negative	1,404 (36.0%)
**Tumor HER2 status**	
Positive	495 (13.2%)
Negative	3,242 (86.8%)
**Tumor IHC subtype**	
Luminal A	2,802 (75.0%)
Luminal B	324 (8.7%)
HER2 enriched	171 (4.6%)
Triple-negative	440 (11.8%)
**Surgery**	
Lumpectomy	2,344 (60.1%)
Mastectomy	1,441 (36.9%)
None	117 (3.0%)
**Chemotherapy**	
Yes	1,829 (47.0%)
No	2,061 (53.0%)
**Radiotherapy**	
Yes	1,731 (44.4%)
No	2,171 (55.6%)
**Hormonal therapy**	
Yes	2,904 (74.9%)
No	972 (25.1%)

**TABLE 2:** Survival events in the Pathways Study (*n* = 3,902)

	*n* (%)
**Survival outcome**	
Total death	762 (19.5%)
Breast cancer–specific death	352 (9.02%)
Recurrence	504 (12.9%)
Total second primary cancer	419 (10.7%)
Disease-free survival	1,191 (30.5%)
Invasive breast cancer event	645 (16.5%)
Total breast cancer event	685 (17.6%)
Cardiovascular death	133 (3.41%)

### Associations of PGS for Metabolic Traits and Breast Cancer Outcomes

**FIGURE 1:**
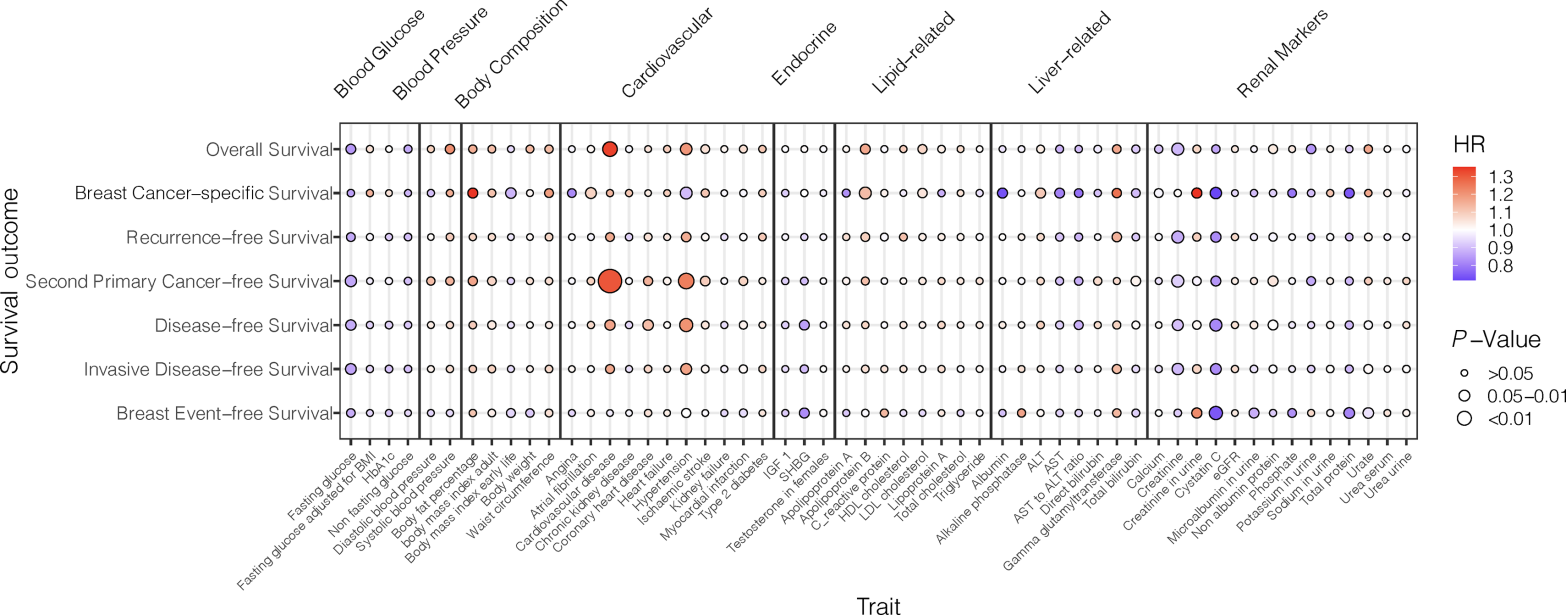
A dot plot summarizes the associations in the analysis of 3,902 women with breast cancer. The *x*-axis represents the 55 PGS for metabolic traits and the *y*-axis represents the seven survival outcomes. Each dot represents a PGS tertile (T3 vs. T1)-survival outcome association. The size of the point is inversely proportional to its *P* value. *P* values are from trend tests by treating T1, T2, and T3 as ordinal variables. The color of the point represents the HR. Bluer colors represent *HR* < 1.0, and more red colors represent *HR* > 1.0.

Figure 1 summarizes the associations of PGS of the 55 metabolic traits with the seven breast outcomes in all the racial and ethnic groups combined after adjustment for covariates. PGS for three of the traits tested were associated with one or more survival outcomes at a nominal significance level of *P* < 0.05, which included CVD, serum cystatin C levels, and hypertension. When Bonferroni correction was applied for testing 55 PGS, no associations met the *P* < 0.0009 threshold. The analyses were then repeated among 2,696 White patients, where the associations remained largely consistent (Supplementary Fig. S1; Supplementary Table S3).

**FIGURE 2:**
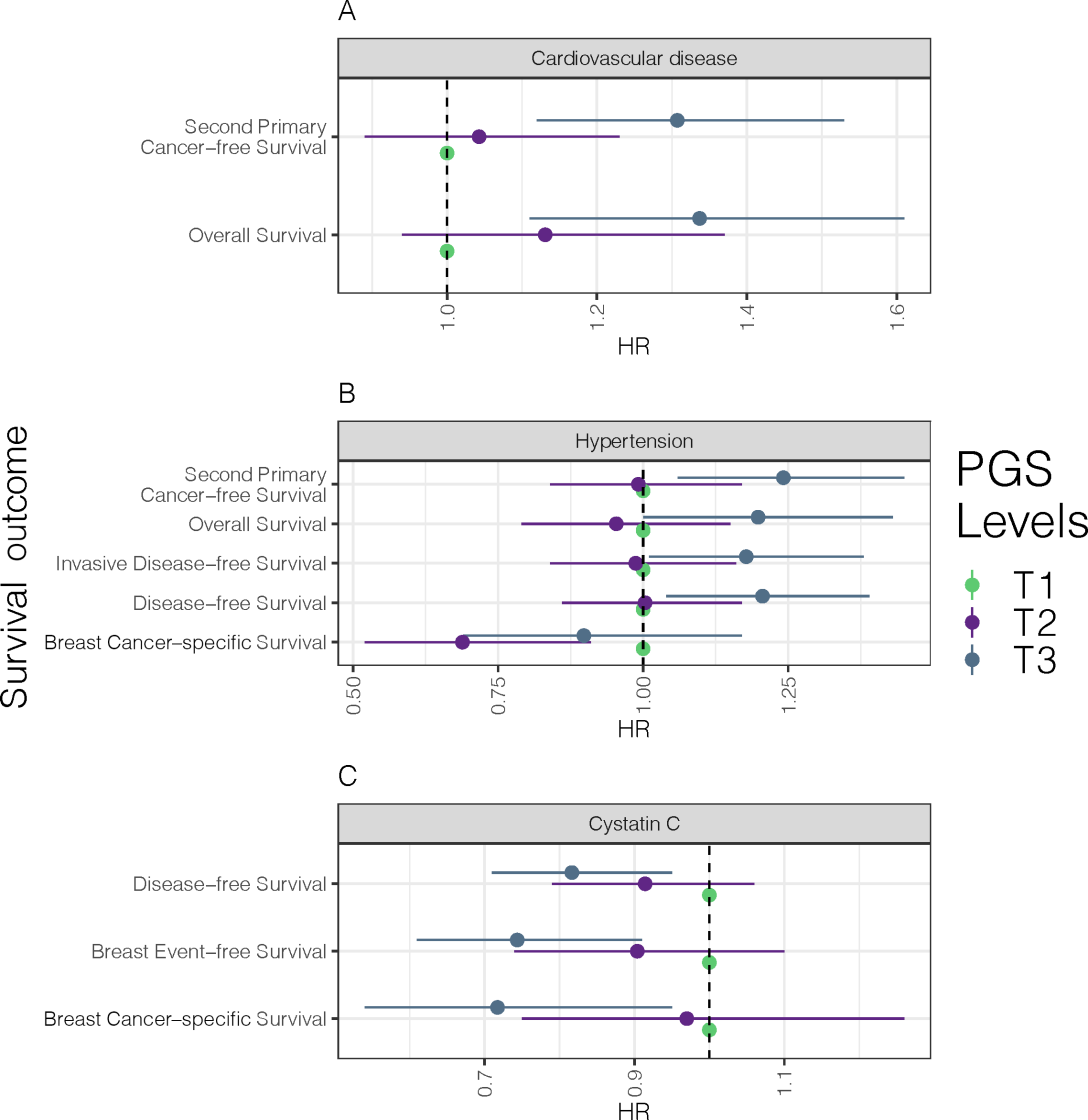
Three forest plots depict the HRs for nominally significant (*P* < 0.05) survival outcome associations with PGS for three different traits: CVD (**A**), serum cystatin C (**B**), and hypertension (**C**). Each point on the plot represents a test between the PGS-tertile and breast cancer-related survival outcome. Green represents tertile 1. Violet represents tertile 2. Blue represents tertile 3. The *y*-axis shows the survival outcome, and the *x*-axis represents the HR. Horizontal lines represent 95% CIs from the Cox regression. Associations with *P* < 0.05 and HR CIs overlapping with one were removed from the plots.

Figure 2 shows the significant associations of the three PGS in association with survival outcomes. In comparison with the first tertile (T1), the third tertile (T3) of the CVD PGS was associated with shorter overall survival (HR = 1.34, 95% CI = 1.11–1.61) and second primary cancer–free survival (HR = 1.31, 95% CI = 1.12–1.53; Fig. 2A). The PGS for serum cystatin C levels was associated with longer disease-free survival (T3: HR = 0.82, 95% CI = 0.71–0.95), breast event-free survival (T3: HR = 0.74, 95% CI = 0.61–0.91), and breast cancer–specific survival (T3: HR = 0.72, 95% CI = 0.54–0.95; Fig. 2B). Finally, the PGS for hypertension was associated with shorter second primary cancer–free survival (T3: HR = 1.24, 95% CI = 1.06–1.45), overall survival (T3: HR = 1.20, 95% CI = 1.00–1.43), invasive disease-free survival (T3: HR = 1.18, 95% CI = 1.01–1.38), and disease-free survival (T3: HR = 1.21, 95% CI = 1.04–1.39). Notably, T2 of this PGS was also associated with shorter breast cancer–specific survival (T2: HR = 0.69, 95% CI = 0.52–0.91; Fig. 2C). When death due to cardiovascular causes was considered as a competing risk in the Cox proportional hazards model, the above results remain essentially unchanged.

### Associations of PGS with Metabolic Traits and Breast Cancer Outcomes Stratified by Tumor ER Status

**FIGURE 3:**
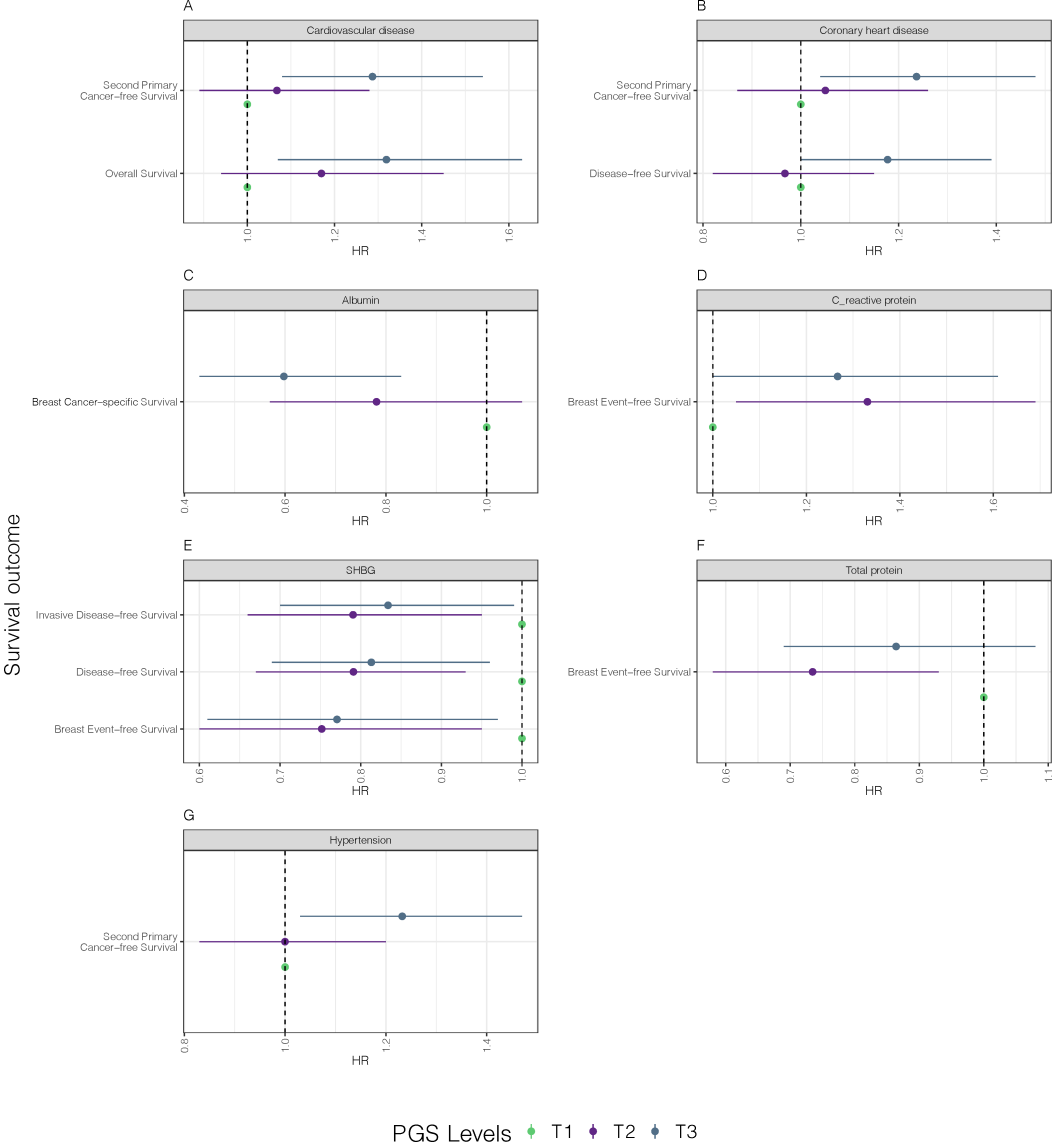
Seven forest plots depict the HRs in the ER^+^ group for nominally significant (*P* < 0.05) survival outcome associations with PGS for seven different traits: CVD (**A**), coronary heart disease (**B**), serum albumin (**C**), C-reactive protein (**D**), SHBG (**E**), total serum protein (**F**), and hypertension (**G**). Each point on the plot represents a test between the PGS-tertile and breast cancer–related survival outcome. Green represents tertile 1. Violet represents tertile 2. Blue represents tertile 3. The *y*-axis shows the survival outcome, and the *x*-axis represents the HR. Horizontal lines represent 95% CIs from the Wald test. Associations with *P* < 0.05, but HR CIs overlapping with one were removed from the plots.

As tumor ER status defines two distinct breast cancer subtypes for both prognosis and treatment, we stratified the analysis of the 55 PGS for metabolic traits by ER status (Supplementary Fig. S2). Figure 3 displays the associations of seven metabolic trait PGS with breast cancer outcomes for individuals with ER^+^ tumors. Compared with T1, T3 of the PGS for CVD was associated with shorter second primary cancer–free survival (HR = 1.22, 95% CI = 1.03–1.45) and shorter overall survival (HR = 1.27, 95% CI = 1.03–1.45; Fig. 3A). T3 for coronary heart disease PGS was associated with shorter second primary cancer–free survival (HR = 1.24, 95% CI = 1.04–1.48) and disease-free survival (HR = 1.18, 95% CI = 1.00–1.39; Fig. 3B). T3 for PGS of serum albumin was associated with longer breast cancer–specific survival (HR = 0.60, 95% CI = 0.43–0.83; Fig. 3C). PGS for serum C-reactive protein was associated with shorter breast event-free survival (T2: HR = 1.33, 95% CI = 1.05–1.69; T3: HR = 1.27, 95% CI = 1.00–1.61; Fig. 3D). PGS for sex hormone binding globulin (SHBG) was associated with longer invasive disease-free survival (T3: HR = 0.84, 95% CI = 0.70–0.99), disease-free survival (T3: HR = 0.81, 95% CI = 0.69–0.96), and breast event-free survival (T3: HR = 0.77, 95% CI = 0.61–0.97; Fig. 3E). PGS for total serum protein was associated with longer breast event-free survival (T2: HR = 0.73, 95% CI = 0.58–0.93; Fig. 3F). Finally, PGS for hypertension was associated with shorter second primary cancer–free survival (T3: HR = 1.23, 95% CI = 1.03–1.47; Fig. 3G).

**FIGURE 4:**
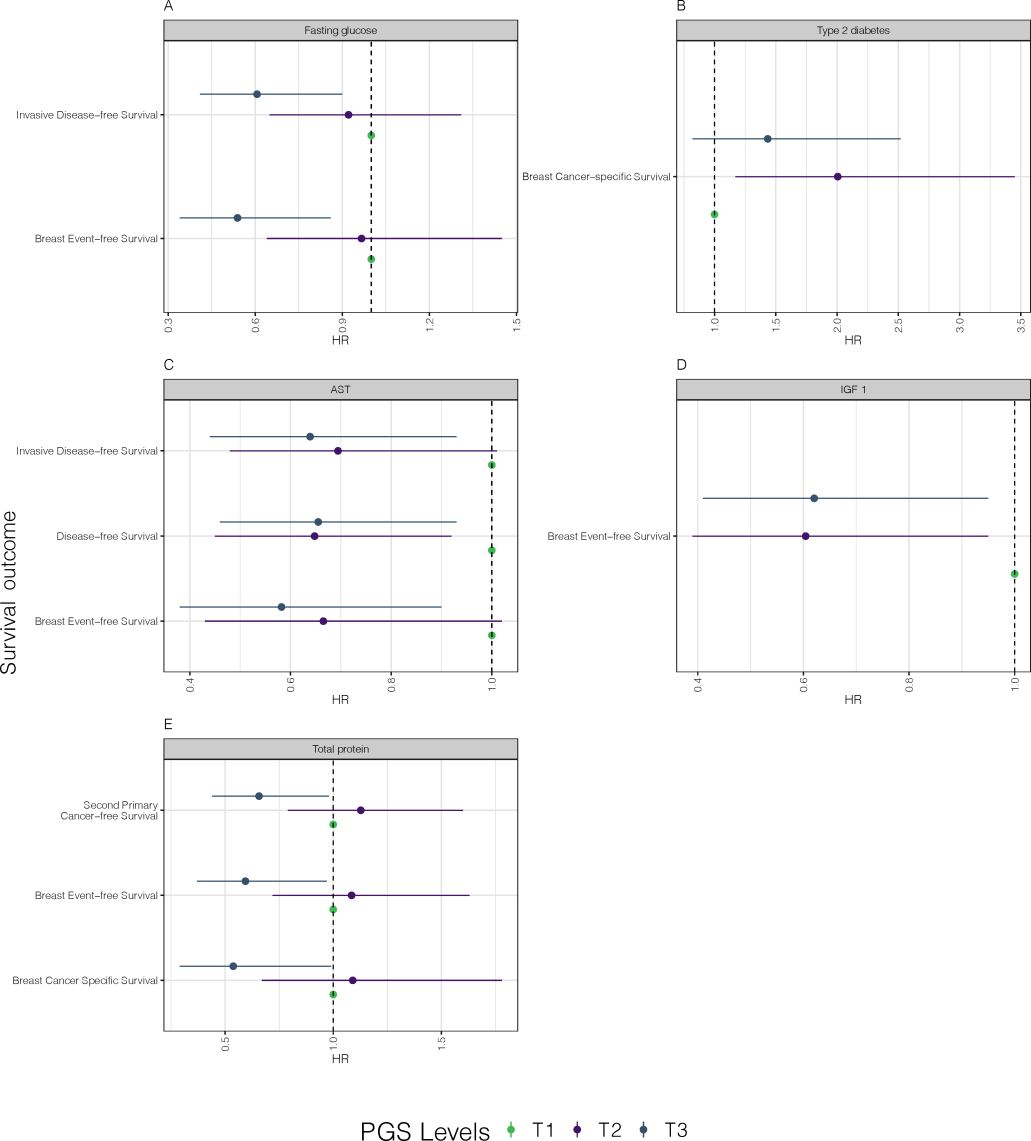
Five forest plots depict the HRs calculated in the ER^−^ group for nominally significant (*P* < 0.05) survival outcome associations with PGS for five different traits: serum fasting glucose (**A**), type 2 diabetes (**B**), AST (**C**), IGF-1 (**D**), and total serum protein (**E**). Each point on the plot represents a test between the PGS-tertile and breast cancer–related survival outcome. Green represents tertile 1. Violet represents tertile 2. Blue represents tertile 3. The *y*-axis shows the survival outcome, and the *x*-axis represents the HR. Horizontal lines represent 95% CIs from the Wald test. Associations with *P* < 0.05, but HR CIs overlapping with one were removed from the plot.

Figure 4 shows the associations in patients with breast cancer with ER^−^ tumors. T3 of PGS for serum fasting glucose levels was associated with longer invasive disease-free survival (HR = 0.61, 95% CI = 0.41–0.90) and breast event-free survival (HR = 0.54, 95% CI = 0.34–0.86; Fig. 4A). Type 2 diabetes PGS was associated with shorter breast cancer–specific survival (T2: HR = 2.01, 95% CI = 1.17–3.45; Fig. 4B). T3 of PGS for aspartate aminotransferase (AST) was associated with invasive disease-free survival (HR = 0.64, CI = 0.44–0.93), disease-free survival (HR = 0.66, 0.46–0.93), and breast event-free survival (HR = 0.58, CI = 0.38–0.90; Fig. 4C). T3 of PGS for serum insulin growth factor-1 (IGF-1) was associated with longer breast event-free survival (HR = 0.62, 95% CI = 0.41–0.95; Fig. 4D). Finally, T3 of PGS for total serum protein was associated with longer second primary cancer–free survival (HR = 0.66, 95% CI = 0.44–0.98), breast event-free survival (HR = 0.59, 95% CI = 0.37–0.97), and breast cancer–specific survival (HR = 0.54, 95% CI = 0.29–0.99; Fig. 4E).

## Discussion

In the largest, most contemporary Mendelian randomization analysis for metabolic traits and breast cancer survival outcomes, we identified associations between PGS for CVD, cystatin C levels, and hypertension in relation to breast cancer survival outcomes at a nominal statistical significance level. The associations differed when stratified by tumor ER status, with total serum protein as the only trait to be nominally significantly associated with survival in both ER^+^ and ER^−^ cancer.

There is growing evidence to link certain metabolic traits with breast cancer risk (15, 19). Chen and colleagues (2022) used Mendelian randomization to examine the relationship between 23 risk factors and risk for developing breast cancer in individuals from the Breast Cancer Association Consortium (20). While this study focused on breast cancer risk, we explored 55 different metabolic traits and breast cancer survival outcomes. Notably, our study contributes to the existing literature in several aspects. First, our study evaluated the relationships between these metabolic traits and breast cancer survival outcomes. While other studies on breast cancer risk may have larger sample sizes, the Pathways cohort is one of the largest and most comprehensively characterized cohorts of breast cancer survivors with respect to tumor pathology, survival outcomes, and demographic and lifestyle risk factors. Second, our study took an agnostic approach to investigating all metabolic traits with an established genetic prediction score rather than selectively investigating previously suspected risk factors. This agnostic approach allowed us to confirm previous findings while also enabling identification of new metabolic traits that may be associated with breast cancer survival outcomes. One of the associations supported by our data is the finding that genetically predicted serum SHBG is inversely associated with disease-free survival, breast event-free survival, and invasive disease-free survival for women with ER^+^ tumors (20). Beyond SHBG, Chen and colleagues found associations between fasting insulin levels and increased risk for breast cancer. Fasting glucose levels were also inversely associated with invasive disease-free and breast event-free survival; however, genetic risk for diabetes was associated with increased risk for breast cancer–specific death. Metabolic traits such as type 2 diabetes and BMI have previously been associated with breast cancer survival outcomes (21), yet our study did not detect such an association. Reasons for this may include suboptimal performance of PGS and limited sample size as we outline below. Beyond these, there is a possibility that the original associations of type 2 diabetes and BMI with survival outcomes are not causal. In the context of previous findings, our results emphasize the need to further investigate glucose metabolism and diabetes in relation to breast cancer outcomes.

With respect to notable findings in our analysis, we highlight the associations of breast cancer survival with CVD, hypertension, and cystatin C levels. First, we found associations of genetic risk for CVD with poorer overall survival and second primary cancer–free survival in patients with breast cancer. Given that CVD is the leading cause of death in the United States and in breast cancer survivors, this finding is particularly surprising (22–24). Women with breast cancer have increased incidence of CVD events, CVD-related mortality, and all-cause mortality compared with women with breast cancer (25). This complex inter-relationship between the genetic risk of CVD and cardiotoxic effects of breast cancer would warrant further investigation in the setting like the Pathways Heart Study, which has established that certain breast cancer treatment increased risk of cardiometabolic conditions and CVDs (25, 26). This study showed that treatment with anthracyclines and trastuzumab increased risk of ischemic heart disease and heart failure in women with breast cancer. In addition to identifying a relationship between CVD PGS and breast cancer outcome, our analyses revealed that genetic risk for hypertension was associated with poor second primary cancer–free survival and overall death. For both CVD and hypertension, these associations with outcome were not substantially changed after considering cardiovascular causes of death as competing risks in the Cox proportional hazards model. Given that hypertension contributes to CVD, a shared genetic architecture between these two diseases may have a third effect on breast cancer outcomes.

Finally, we observed increased genetically predicted cystatin C levels to be associated with better disease-free, breast event-free, and breast cancer–specific survival. In addition to being a commonly-tested marker for renal function, cystatin C is an endogenous inhibitor of cysteine cathepsin proteases, commonly dysregulated in tumorigenesis (27). Expression of cystatin C, also a target of p53, has previously been shown to be downregulated in breast cancer cells with p53 mutations (28). By decreasing the levels of cystatin C, cysteine cathepsin proteases are disinhibited. Elevated cathepsin levels have previously been significantly associated with poor prognosis in breast, lung, head and neck, and colorectal cancers (29, 30). Our findings support this hypothesis given that PGS for elevated cystatin C levels were associated with better breast cancer survival outcomes.

Limitations should be noted in this study, which include sample size, particularly for non-White populations, lack of confirmatory analyses in replication cohorts, and the potential variation in accuracy of the PGS models by race and ethnicity. Although the Pathways Study is one of the largest existing, well-characterized cohorts of patients with breast cancer, with 3,902 patients included in this analysis, concerns remain regarding sample size for this study of Mendelian randomization. Identifying an appropriate independent cohort to replicate our findings is challenging given the scarcity of large, deeply phenotyped breast cancer survivorship cohorts. A replication cohort would be informative to support our findings, especially those that challenge suppositions regarding outcomes in women diagnosed with breast cancer. Moreover, the PGS models used in our analyses were generally developed in European ancestry populations. When possible, we tried to control for this potential issue by using ancestry-matched polygenic prediction models for different ethnicities in the Pathways cohort. However, for models built from only European populations, the transferability of these models to other ancestral populations is limited (31). Importantly, the PGS models used for hypertension, CVD, type 2 diabetes, and coronary heart disease were trained and tested in populations of European ancestry. The performance of these PGS in populations of non-European ancestry like those in the Pathways Study has not been independently validated. Going forward, this issue will need to be addressed by increasing the number of individuals of non-European ancestries when training PGS models.

In summary, by comprehensively examining PGS for 55 metabolic traits in a large population of patients with breast cancer, we observed three traits for which their genetic scores were consistently associated with breast cancer survival outcomes, including complimentary associations of cardiovascular and hypertension PGS with shorter patient survival outcomes. Our findings highlight the complexity between metabolic syndrome and breast cancer prognosis; therefore, this relationship warrants further investigation in future studies to better understand how metabolic syndrome, and notably cardiovascular traits, influence breast cancer outcomes. The results may facilitate the development of novel prognostic markers for breast cancer outcomes.

## Supplementary Material

Supplementary Table 1 Polygenic score models pulled from the polygenic score catalog

Supplementary Table 2 Statistically-significant findings from analysis of 2,696 White individuals

Supplementary Table 3 Time-varying covariates for Cox proportional hazard model

Supplementary Figure 1 Dot plot of findings from analysis of 2,696 White individuals

Supplementary Figure 2 Dot plot of statistically-significant findings stratified by tumor estrogen receptor (ER) status in whole analytic population, with 3,260 ER+ and 640 ER- individuals
