# Supplementary material for: A Mendelian Randomization Analysis of 55 Genetically Predicted Metabolic Traits with Breast Cancer Survival Outcomes in the Pathways Study
Source: Cancer Res Commun. 2023 Jun 22;3(6):1104–12. doi: 10.1158/2767-9764.CRC-23-0047 (PMC10286812; doi:10.1158/2767-9764.CRC-23-0047)
Supplement: Supplementary Table 1 — Polygenic score models pulled from the polygenic score catalog [file crc-23-0047-s01.docx]

**Supplemental Table 1. Polygenic score models pulled from the polygenic score catalog**

| **Trait** | **Number of variants** | **Performance in Europeans** | **Performance in Africans** | **Performance in East Asians** | **PGS #** | **Reference (PMID)** | **Percent Variant Matched** |
| --- | --- | --- | --- | --- | --- | --- | --- |
| Total cholesterol | 17,204 | R2=0.264 | R2=0.128 | R2=0.172 | PGS000677 | 33462484 | 95.12 |
| Triglyceride | 16,003 | R2=0.232 | R2=0.106 | R2=0.176 | PGS000699 | 33462484 | 94.98 |
| LDL cholesterol | 16,184 | R2=0.264 | R2=0.134 | R2=0.140 | PGS000688 | 33462484 | 95.28 |
| HDL cholesterol | 25,069 | R2=0.361 | R2=0.175 | R2=0.315 | PGS000686 | 33462484 | 95.35 |
| Apolipoprotein A | 19,324 | R2=0.315 | R2=0.149 | R2=0.320 | PGS000671 | 33462484 | 95.38 |
| Apolipoprotein B | 18,666 | R2=0.302 | R2=0.157 | R2=0.177 | PGS000672 | 33462484 | 95.19 |
| Lipoprotein A | 8,308 | R2=0.516 | R2=0.008 | R2=0.062 | PGS000689 | 33462484 | 94.38 |
| C-reactive protein | 17,378 | R2=0.160 | R2=0.077 | R2=0.107 | PGS000675 | 33462484 | 95.11 |
| Systolic blood pressure | 15,481 | R2=0.194 | R2=0.103 | R2=0.229 | PGS001134 | 35324888 | 95.39 |
| Diastolic blood pressure | 14,103 | R2=0.085 | R2= 0.010 | R2=0.109 | PGS001133 | 35324888 | 95.16 |
| Non-fasting glucose | 3,313 | R2=0.070 | R2=0.052 | R2=0.082 | PGS000684 | 33462484 | 92.39 |
| Fasting glucose | 31 | R2=0.037 | NA | NA | PGS000305 | 32527150 | 100 |
| Fasting glucose adjusted for BMI | 19 | R2=0.010 | NA | NA | PGS000306 | 32527150 | 100 |
| HbA1c | 14,658 | R2=0.173 | R2=0.080 | R2=0.170 | PGS000685 | 33462484 | 94.68 |
| Albumin | 11,912 | R2=0.133 | R2=0.101 | R2=0.089 | PGS000669 | 33462484 | 95.28 |
| ALT | 12,076 | R2=0.204 | R2=0.176 | R2=0.156 | PGS000668 | 33462484 | 95.16 |
| AST | 12,829 | R2=0.156 | R2=0.103 | R2=0.083 | PGS000673 | 33462484 | 94.75 |
| AST to ALT ratio | 15,548 | R2=0.179 | R2=0.100 | R2=0.160 | PGS000674 | 33462484 | 95.13 |
| Gamma glutamyltransferase | 17,323 | R2=0.266 | R2=0.121 | R2=0.161 | PGS000683 | 33462484 | 95.1 |
| Direct bilirubin | 3,104 | R2=0.343 | R2=0.279 | R2=0.158 | PGS000681 | 33462484 | 93.49 |
| Total bilirubin | 1,159 | R2=0.444 | R2=0.387 | R2=0.216 | PGS000697 | 33462484 | 93.87 |
| Alkaline phosphatase | 18,328 | R2=0.291 | R2=0.111 | R2=0.255 | PGS000670 | 33462484 | 95.37 |
| Creatinine | 21,027 | R2=0.416 | R2=0.371 | R2=0.443 | PGS000678 | 33462484 | 95.34 |
| Creatinine in urine | 5,469 | R2=0.161 | R2=0.112 | R2=0.165 | PGS000679 | 33462484 | 93.98 |
| Cystatin C | 24,487 | R2=0.305 | R2=0.177 | R2=0.289 | PGS000680 | 33462484 | 95.26 |
| eGFR | 17,467 | R2=0.255 | R2=0.178 | R2=0.211 | PGS000682 | 33462484 | 95.03 |
| Microalbumin in urine | 111 | R2=0.021 | R2=0.023 | R2=0.001 | PGS000690 | 33462484 | 77.48 |
| Non-albumin protein | 18,670 | R2=0.128 | R2=0.071 | R2=0.089 | PGS000691 | 33462484 | 95.3 |
| Phosphate | 12,448 | R2=0.238 | R2=0.128 | R2=0.203 | PGS000692 | 33462484 | 94.79 |
| Potassium in urine | 2,423 | R2=0.048 | R2=0.030 | R2=0.019 | PGS000693 | 33462484 | 93.19 |
| Sodium in urine | 5,833 | R2=0.110 | R2=0.046 | R2=0.097 | PGS000695 | 33462484 | 93.86 |
| Total protein | 16,420 | R2=0.117 | R2=0.044 | R2=0.093 | PGS000698 | 33462484 | 95.24 |
| Urate | 20,171 | R2=0.421 | R2=0.295 | R2=0.338 | PGS000700 | 33462484 | 95.27 |
| Urea - serum | 12,351 | R2=0.158 | R2=0.130 | R2=0.129 | PGS000701 | 33462484 | 94.68 |
| Urea - urine | 10,852 | R2=0.053 | R2=0.017 | R2=0.041 | PGS000971 | 35324888 | 94.66 |
| Calcium | 12,334 | R2=0.115 | R2=0.073 | R2=0.109 | PGS000676 | 33462484 | 94.78 |
| IGF-1 | 23,443 | R2=0.223 | R2=0.115 | R2=0.177 | PGS000687 | 33462484 | 95.31 |
| SHBG | 19,328 | R2=0.355 | R2=0.227 | R2=0.318 | PGS000694 | 33462484 | 95.62 |
| Testosterone in females | 7,168 | R2=0.180 | NA | NA | PGS000322 | 32873964 | 96.92 |
| body mass index adult | 27,126 | R2=0.131 | R2=0.063 | R2=0.166 | PGS001228 | 35324888 | 95.56 |
| Body mass index early life | 295 | AUROC=0.64 | NA | NA | PGS000716 | 33276378 | 99.66 |
| Waist circumference | 25,538 | R2=0.305 | R2=0.049 | R2=0.315 | PGS001227 | 35324888 | 95.41 |
| Body fat percentage | 27,396 | R2=0.512 | R2=0.586 | R2=0.440 | PGS001101 | 35324888 | 95.46 |
| Body weight | 31,222 | R2=0.331 | R2=0.062 | R2=0.364 | PGS001230 | 35324888 | 95.38 |
| Chronic kidney disease | 1,958,860 | ?= -0.9 [1.45, -0.36] | NA | NA | PGS000728 | 34750571 | 99.26 |
| Kidney failure | 183,272 | AUROC=0.561 | NA | NA | PGS000708 | 33462484 | 91.96 |
| Type 2 diabetes | 6,437,380 | C-index=0.845 | NA | NA | PGS000330 | 32273609 | 97.5 |
| Heart failure | 183,287 | AUROC=0.532 | NA | NA | PGS000709 | 33462484 | 92.16 |
| Myocardial infarction | 183,566 | AUROC=0.593 | NA | NA | PGS000710 | 33462484 | 92.18 |
| Angina | 183,692 | AUROC=0.593 | NA | NA | PGS000703 | 33462484 | 92.2 |
| Hypertension | 1,098,015 | HR=2.12 [1.99, 2.25] | NA | NA | PGS000900 | 34397277 | 91.29 |
| Atrial fibrillation | 6,183,494 | C-index=0.751 | NA | NA | PGS000331 | 32273609 | 97.45 |
| Coronary heart disease | 6,423,165 | C-index=0.832 | NA | NA | PGS000329 | 32273609 | 97.68 |
| Ischaemic stroke | 3,225,583 | C-index=0.585 [0.574, 0.595] | NA | NA | PGS000039 | 31862893 | 99.26 |
| Cardiovascular disease | 297,862 | C-index*=0.75 [0.75, 0.76] | NA | NA | PGS000117 | 32068818 | 99.64 |

**Footnote:** A table that presents source information on the 55 polygenic score models used to generate polygenic scores for each individual in our study. For each polygenic score model, the table includes the number of SNPs used to predict polygenic score, the model’s performance in different ancestries, its PGS number corresponding to the PGS Catalog, the PubMed ID of the model’s original paper, and the proportion of the variants in the model that were used to calculate PGS from the Pathways genotype data.
