# Supplementary material for: A Mendelian Randomization Analysis of 55 Genetically Predicted Metabolic Traits with Breast Cancer Survival Outcomes in the Pathways Study
Source: Cancer Res Commun. 2023 Jun 22;3(6):1104–12. doi: 10.1158/2767-9764.CRC-23-0047 (PMC10286812; doi:10.1158/2767-9764.CRC-23-0047)
Supplement: Supplementary Table 2 — Statistically-significant findings from analysis of 2,696 White individuals [file crc-23-0047-s02.docx]

**Supplemental Table 2. Statistically-significant findings from analysis of 2,696 White individuals**

| **PGS Trait** | **Survival Outcome** | **Adjusted P-value** | **T2 HR (95% CI)** | **T3 HR (95% CI)** |
| --- | --- | --- | --- | --- |
| Cardiovascular disease | Second Primary Cancer-free Survival | 0.004 | 0.78 (0.65, 0.93) | 0.76 (0.64, 0.91) |
| Hypertension | Second Primary Cancer-free Survival | 0.007 | 0.97 (0.80, 1.16) | 1.26 (1.05, 1.50) |
| Alkaline phosphatase | Breast Event-free Survival | 0.007 | 1.18 (0.92, 1.52) | 1.46 (1.15, 1.85) |
| Hypertension | Disease-free Survival | 0.010 | 0.96 (0.81, 1.14) | 1.22 (1.03, 1.44) |
| Cystatin C | Breast Cancer Specific Survival | 0.013 | 0.88 (0.64, 1.21) | 0.61 (0.44, 0.86) |
| Cardiovascular disease | Overall Survival | 0.020 | 0.86 (0.70, 1.05) | 0.74 (0.60, 0.91) |
| Hypertension | Invasive Disease-free Survival | 0.027 | 0.96 (0.79, 1.16) | 1.21 (1.01,1.45) |
| Creatinine | Overall Survival | 0.028 | 1.06 (0.86, 1.30) | 0.80 (0.65,1) |
| Creatinine | Recurrence-free Survival | 0.030 | 1.04 (0.86, 1.25) | 0.82 (0.67,0.99) |
| Angina | Breast Cancer Specific Survival | 0.032 | 0.67 (0.48, 0.93) | 0.71 (0.52,0.98) |
| Hypertension | Overall Survival | 0.042 | 0.99 (0.80, 1.24) | 1.25 (1.02,1.54) |

**Footnote:** This table shows the covariates used for all 385 tests. “Trait” refers to the PGS for the respective metabolic trait. “Survival Outcome” refers to one of the seven survival outcomes considered. “Time_Varying_Covariates” refers to a list of covariates that were considered time-varying for a given test between PGS and survival outcome.
