# Supplementary material for: A Mendelian Randomization Analysis of 55 Genetically Predicted Metabolic Traits with Breast Cancer Survival Outcomes in the Pathways Study
Source: Cancer Res Commun. 2023 Jun 22;3(6):1104–12. doi: 10.1158/2767-9764.CRC-23-0047 (PMC10286812; doi:10.1158/2767-9764.CRC-23-0047)
Supplement: Supplementary Table 3 — Time-varying covariates for Cox proportional hazard model [file crc-23-0047-s03.docx]

**Supplemental Table S3. Time-varying covariates for Cox proportional hazard model.**

| **Trait** | **Survival Outcome** | **Time-Varying Covariates** |
| --- | --- | --- |
| Cardiovascular disease | Overall Survival | AJCC Stage, Tumor Grade, IHC Subtype, Hormonal Therapy, Chemotherapy, Radiation |
| Cardiovascular disease | Breast Cancer Specific Survival | AJCC Stage, Tumor Grade, IHC Subtype, Hormonal Therapy, Chemotherapy |
| Cardiovascular disease | Recurrence-free Survival | Race/Ethnicity, AJCC Stage, Tumor Grade, IHC Subtype, Hormonal Therapy, Chemotherapy, Radiation, Surgery |
| Cardiovascular disease | Disease-free Survival | Race/Ethnicity, AJCC Stage, Tumor Grade, IHC Subtype, Hormonal Therapy, Chemotherapy, Surgery |
| Cardiovascular disease | Invasive Disease-free Survival | Race/Ethnicity, AJCC Stage, Tumor Grade, IHC Subtype, Hormonal Therapy, Chemotherapy, Surgery |
| Cardiovascular disease | Second Primary Cancer-free Survival | Tumor Grade, IHC Subtype, Hormonal Therapy, Chemotherapy |
| Cardiovascular disease | Breast Event-free Survival | AJCC Stage, Tumor Grade, IHC Subtype, Hormonal Therapy |
| Ischaemic stroke | Overall Survival | AJCC Stage, Tumor Grade, IHC Subtype, Hormonal Therapy, Chemotherapy, Radiation |
| Ischaemic stroke | Breast Cancer Specific Survival | AJCC Stage, Tumor Grade, IHC Subtype, Hormonal Therapy, Chemotherapy |
| Ischaemic stroke | Recurrence-free Survival | Race/Ethnicity, AJCC Stage, Tumor Grade, IHC Subtype, Hormonal Therapy, Chemotherapy, Radiation, Surgery |
| Ischaemic stroke | Disease-free Survival | Race/Ethnicity, AJCC Stage, Tumor Grade, IHC Subtype, Hormonal Therapy, Chemotherapy, Surgery |
| Ischaemic stroke | Invasive Disease-free Survival | Race/Ethnicity, AJCC Stage, Tumor Grade, IHC Subtype, Hormonal Therapy, Chemotherapy, Surgery |
| Ischaemic stroke | Second Primary Cancer-free Survival | Tumor Grade, IHC Subtype, Hormonal Therapy, Chemotherapy |
| Ischaemic stroke | Breast Event-free Survival | AJCC Stage, Tumor Grade, IHC Subtype, Hormonal Therapy |
| Fasting glucose | Overall Survival | AJCC Stage, Tumor Grade, IHC Subtype, Hormonal Therapy, Chemotherapy, Radiation, Surgery |
| Fasting glucose | Breast Cancer Specific Survival | AJCC Stage, Tumor Grade, IHC Subtype, Hormonal Therapy, Chemotherapy |
| Fasting glucose | Recurrence-free Survival | Race/Ethnicity, AJCC Stage, Tumor Grade, IHC Subtype, Hormonal Therapy, Chemotherapy, Radiation, Surgery |
| Fasting glucose | Disease-free Survival | Race/Ethnicity, AJCC Stage, Tumor Grade, IHC Subtype, Hormonal Therapy, Chemotherapy, Surgery |
| Fasting glucose | Invasive Disease-free Survival | Race/Ethnicity, AJCC Stage, Tumor Grade, IHC Subtype, Hormonal Therapy, Chemotherapy, Surgery |
| Fasting glucose | Second Primary Cancer-free Survival | Tumor Grade, IHC Subtype, Hormonal Therapy, Chemotherapy |
| Fasting glucose | Breast Event-free Survival | AJCC Stage, Tumor Grade, IHC Subtype, Hormonal Therapy |
| Fasting glucose adjusted for BMI | Overall Survival | AJCC Stage, Tumor Grade, IHC Subtype, Hormonal Therapy, Chemotherapy, Radiation |
| Fasting glucose adjusted for BMI | Breast Cancer Specific Survival | AJCC Stage, Tumor Grade, IHC Subtype, Hormonal Therapy, Chemotherapy |
| Fasting glucose adjusted for BMI | Recurrence-free Survival | Race/Ethnicity, AJCC Stage, Tumor Grade, IHC Subtype, Hormonal Therapy, Chemotherapy, Radiation, Surgery |
| Fasting glucose adjusted for BMI | Disease-free Survival | Race/Ethnicity, AJCC Stage, Tumor Grade, IHC Subtype, Hormonal Therapy, Chemotherapy, Surgery |
| Fasting glucose adjusted for BMI | Invasive Disease-free Survival | Race/Ethnicity, AJCC Stage, Tumor Grade, IHC Subtype, Hormonal Therapy, Chemotherapy, Surgery |
| Fasting glucose adjusted for BMI | Second Primary Cancer-free Survival | Tumor Grade, IHC Subtype, Hormonal Therapy, Chemotherapy |
| Fasting glucose adjusted for BMI | Breast Event-free Survival | AJCC Stage, Tumor Grade, IHC Subtype, Hormonal Therapy |
| Testosterone in females | Overall Survival | AJCC Stage, Tumor Grade, IHC Subtype, Hormonal Therapy, Chemotherapy, Radiation |
| Testosterone in females | Breast Cancer Specific Survival | AJCC Stage, Tumor Grade, IHC Subtype, Hormonal Therapy, Chemotherapy |
| Testosterone in females | Recurrence-free Survival | Race/Ethnicity, AJCC Stage, Tumor Grade, IHC Subtype, Hormonal Therapy, Chemotherapy, Radiation, Surgery |
| Testosterone in females | Disease-free Survival | Race/Ethnicity, AJCC Stage, Tumor Grade, IHC Subtype, Hormonal Therapy, Chemotherapy, Surgery |
| Testosterone in females | Invasive Disease-free Survival | Race/Ethnicity, AJCC Stage, Tumor Grade, IHC Subtype, Hormonal Therapy, Chemotherapy, Surgery |
| Testosterone in females | Second Primary Cancer-free Survival | Tumor Grade, IHC Subtype, Hormonal Therapy, Chemotherapy |
| Testosterone in females | Breast Event-free Survival | AJCC Stage, Tumor Grade, IHC Subtype, Hormonal Therapy |
| Coronary heart disease | Overall Survival | AJCC Stage, Tumor Grade, IHC Subtype, Hormonal Therapy, Chemotherapy, Radiation |
| Coronary heart disease | Breast Cancer Specific Survival | AJCC Stage, Tumor Grade, IHC Subtype, Hormonal Therapy, Chemotherapy |
| Coronary heart disease | Recurrence-free Survival | Race/Ethnicity, AJCC Stage, Tumor Grade, IHC Subtype, Hormonal Therapy, Chemotherapy, Radiation, Surgery |
| Coronary heart disease | Disease-free Survival | Race/Ethnicity, AJCC Stage, Tumor Grade, IHC Subtype, Hormonal Therapy, Chemotherapy, Surgery |
| Coronary heart disease | Invasive Disease-free Survival | Race/Ethnicity, AJCC Stage, Tumor Grade, IHC Subtype, Hormonal Therapy, Chemotherapy, Surgery |
| Coronary heart disease | Second Primary Cancer-free Survival | Tumor Grade, IHC Subtype, Hormonal Therapy, Chemotherapy |
| Coronary heart disease | Breast Event-free Survival | AJCC Stage, Tumor Grade, IHC Subtype, Hormonal Therapy |
| Type 2 diabetes | Overall Survival | AJCC Stage, Tumor Grade, IHC Subtype, Hormonal Therapy, Chemotherapy, Radiation |
| Type 2 diabetes | Breast Cancer Specific Survival | AJCC Stage, Tumor Grade, IHC Subtype, Hormonal Therapy, Chemotherapy |
| Type 2 diabetes | Recurrence-free Survival | Race/Ethnicity, AJCC Stage, Tumor Grade, IHC Subtype, Hormonal Therapy, Chemotherapy, Radiation, Surgery |
| Type 2 diabetes | Disease-free Survival | Race/Ethnicity, AJCC Stage, Tumor Grade, IHC Subtype, Hormonal Therapy, Chemotherapy, Surgery |
| Type 2 diabetes | Invasive Disease-free Survival | Race/Ethnicity, AJCC Stage, Tumor Grade, IHC Subtype, Hormonal Therapy, Chemotherapy, Surgery |
| Type 2 diabetes | Second Primary Cancer-free Survival | Tumor Grade, IHC Subtype, Hormonal Therapy, Chemotherapy |
| Type 2 diabetes | Breast Event-free Survival | AJCC Stage, Tumor Grade, IHC Subtype, Hormonal Therapy |
| Atrial fibrillation | Overall Survival | AJCC Stage, Tumor Grade, IHC Subtype, Hormonal Therapy, Chemotherapy, Radiation |
| Atrial fibrillation | Breast Cancer Specific Survival | AJCC Stage, Tumor Grade, IHC Subtype, Hormonal Therapy, Chemotherapy |
| Atrial fibrillation | Recurrence-free Survival | Race/Ethnicity, AJCC Stage, Tumor Grade, IHC Subtype, Hormonal Therapy, Chemotherapy, Radiation, Surgery |
| Atrial fibrillation | Disease-free Survival | Race/Ethnicity, AJCC Stage, Tumor Grade, IHC Subtype, Hormonal Therapy, Chemotherapy, Surgery |
| Atrial fibrillation | Invasive Disease-free Survival | Race/Ethnicity, AJCC Stage, Tumor Grade, IHC Subtype, Hormonal Therapy, Chemotherapy, Surgery |
| Atrial fibrillation | Second Primary Cancer-free Survival | Tumor Grade, IHC Subtype, Hormonal Therapy, Chemotherapy |
| Atrial fibrillation | Breast Event-free Survival | AJCC Stage, Tumor Grade, IHC Subtype, Hormonal Therapy |
| ALT | Overall Survival | AJCC Stage, Tumor Grade, IHC Subtype, Hormonal Therapy, Chemotherapy, Radiation |
| ALT | Breast Cancer Specific Survival | AJCC Stage, Tumor Grade, IHC Subtype, Hormonal Therapy, Chemotherapy |
| ALT | Recurrence-free Survival | Race/Ethnicity, AJCC Stage, Tumor Grade, IHC Subtype, Hormonal Therapy, Chemotherapy, Radiation, Surgery |
| ALT | Disease-free Survival | Race/Ethnicity, AJCC Stage, Tumor Grade, IHC Subtype, Hormonal Therapy, Chemotherapy, Surgery |
| ALT | Invasive Disease-free Survival | Race/Ethnicity, AJCC Stage, Tumor Grade, IHC Subtype, Hormonal Therapy, Chemotherapy, Surgery |
| ALT | Second Primary Cancer-free Survival | Tumor Grade, IHC Subtype, Hormonal Therapy, Chemotherapy |
| ALT | Breast Event-free Survival | AJCC Stage, Tumor Grade, IHC Subtype, Hormonal Therapy |
| Albumin | Overall Survival | AJCC Stage, Tumor Grade, IHC Subtype, Hormonal Therapy, Chemotherapy, Radiation |
| Albumin | Breast Cancer Specific Survival | AJCC Stage, Tumor Grade, IHC Subtype, Hormonal Therapy, Chemotherapy |
| Albumin | Recurrence-free Survival | Race/Ethnicity, AJCC Stage, Tumor Grade, IHC Subtype, Hormonal Therapy, Chemotherapy, Radiation, Surgery |
| Albumin | Disease-free Survival | Race/Ethnicity, AJCC Stage, Tumor Grade, IHC Subtype, Hormonal Therapy, Chemotherapy, Surgery |
| Albumin | Invasive Disease-free Survival | Race/Ethnicity, AJCC Stage, Tumor Grade, IHC Subtype, Hormonal Therapy, Chemotherapy, Surgery |
| Albumin | Second Primary Cancer-free Survival | Tumor Grade, IHC Subtype, Hormonal Therapy, Chemotherapy |
| Albumin | Breast Event-free Survival | AJCC Stage, Tumor Grade, IHC Subtype, Hormonal Therapy |
| Alkaline phosphatase | Overall Survival | AJCC Stage, Tumor Grade, IHC Subtype, Hormonal Therapy, Chemotherapy, Radiation |
| Alkaline phosphatase | Breast Cancer Specific Survival | AJCC Stage, Tumor Grade, IHC Subtype, Hormonal Therapy, Chemotherapy |
| Alkaline phosphatase | Recurrence-free Survival | Race/Ethnicity, AJCC Stage, Tumor Grade, IHC Subtype, Hormonal Therapy, Chemotherapy, Radiation, Surgery |
| Alkaline phosphatase | Disease-free Survival | Race/Ethnicity, AJCC Stage, Tumor Grade, IHC Subtype, Hormonal Therapy, Chemotherapy, Surgery |
| Alkaline phosphatase | Invasive Disease-free Survival | Race/Ethnicity, AJCC Stage, Tumor Grade, IHC Subtype, Hormonal Therapy, Chemotherapy, Surgery |
| Alkaline phosphatase | Second Primary Cancer-free Survival | Tumor Grade, IHC Subtype, Hormonal Therapy, Chemotherapy |
| Alkaline phosphatase | Breast Event-free Survival | AJCC Stage, Tumor Grade, IHC Subtype, Hormonal Therapy |
| Apolipoprotein A | Overall Survival | AJCC Stage, Tumor Grade, IHC Subtype, Hormonal Therapy, Chemotherapy, Radiation |
| Apolipoprotein A | Breast Cancer Specific Survival | AJCC Stage, Tumor Grade, IHC Subtype, Hormonal Therapy, Chemotherapy |
| Apolipoprotein A | Recurrence-free Survival | Race/Ethnicity, AJCC Stage, Tumor Grade, IHC Subtype, Hormonal Therapy, Chemotherapy, Radiation, Surgery |
| Apolipoprotein A | Disease-free Survival | Race/Ethnicity, AJCC Stage, Tumor Grade, IHC Subtype, Hormonal Therapy, Chemotherapy, Surgery |
| Apolipoprotein A | Invasive Disease-free Survival | Race/Ethnicity, AJCC Stage, Tumor Grade, IHC Subtype, Hormonal Therapy, Chemotherapy, Surgery |
| Apolipoprotein A | Second Primary Cancer-free Survival | Tumor Grade, IHC Subtype, Hormonal Therapy, Chemotherapy |
| Apolipoprotein A | Breast Event-free Survival | AJCC Stage, Tumor Grade, IHC Subtype, Hormonal Therapy |
| Apolipoprotein B | Overall Survival | AJCC Stage, Tumor Grade, IHC Subtype, Hormonal Therapy, Chemotherapy, Radiation |
| Apolipoprotein B | Breast Cancer Specific Survival | AJCC Stage, Tumor Grade, IHC Subtype, Hormonal Therapy, Chemotherapy |
| Apolipoprotein B | Recurrence-free Survival | Race/Ethnicity, AJCC Stage, Tumor Grade, IHC Subtype, Hormonal Therapy, Chemotherapy, Radiation, Surgery |
| Apolipoprotein B | Disease-free Survival | Race/Ethnicity, AJCC Stage, Tumor Grade, IHC Subtype, Hormonal Therapy, Chemotherapy, Surgery |
| Apolipoprotein B | Invasive Disease-free Survival | Race/Ethnicity, AJCC Stage, Tumor Grade, IHC Subtype, Hormonal Therapy, Chemotherapy, Surgery |
| Apolipoprotein B | Second Primary Cancer-free Survival | Tumor Grade, IHC Subtype, Hormonal Therapy, Chemotherapy |
| Apolipoprotein B | Breast Event-free Survival | AJCC Stage, Tumor Grade, IHC Subtype, Hormonal Therapy |
| AST | Overall Survival | AJCC Stage, Tumor Grade, IHC Subtype, Hormonal Therapy, Chemotherapy, Radiation |
| AST | Breast Cancer Specific Survival | AJCC Stage, Tumor Grade, IHC Subtype, Hormonal Therapy, Chemotherapy |
| AST | Recurrence-free Survival | Race/Ethnicity, AJCC Stage, Tumor Grade, IHC Subtype, Hormonal Therapy, Chemotherapy, Radiation, Surgery |
| AST | Disease-free Survival | Race/Ethnicity, AJCC Stage, Tumor Grade, IHC Subtype, Hormonal Therapy, Chemotherapy, Surgery |
| AST | Invasive Disease-free Survival | Race/Ethnicity, AJCC Stage, Tumor Grade, IHC Subtype, Hormonal Therapy, Chemotherapy, Surgery |
| AST | Second Primary Cancer-free Survival | Tumor Grade, IHC Subtype, Hormonal Therapy, Chemotherapy |
| AST | Breast Event-free Survival | AJCC Stage, Tumor Grade, IHC Subtype, Hormonal Therapy |
| AST to ALT ratio | Overall Survival | AJCC Stage, Tumor Grade, IHC Subtype, Hormonal Therapy, Chemotherapy, Radiation |
| AST to ALT ratio | Breast Cancer Specific Survival | AJCC Stage, Tumor Grade, IHC Subtype, Hormonal Therapy, Chemotherapy |
| AST to ALT ratio | Recurrence-free Survival | Race/Ethnicity, AJCC Stage, Tumor Grade, IHC Subtype, Hormonal Therapy, Chemotherapy, Radiation, Surgery |
| AST to ALT ratio | Disease-free Survival | Race/Ethnicity, AJCC Stage, Tumor Grade, IHC Subtype, Hormonal Therapy, Chemotherapy, Surgery |
| AST to ALT ratio | Invasive Disease-free Survival | Race/Ethnicity, AJCC Stage, Tumor Grade, IHC Subtype, Hormonal Therapy, Chemotherapy, Surgery |
| AST to ALT ratio | Second Primary Cancer-free Survival | Tumor Grade, IHC Subtype, Hormonal Therapy, Chemotherapy |
| AST to ALT ratio | Breast Event-free Survival | AJCC Stage, Tumor Grade, IHC Subtype, Hormonal Therapy |
| C_reactive protein | Overall Survival | AJCC Stage, Tumor Grade, IHC Subtype, Hormonal Therapy, Chemotherapy, Radiation |
| C_reactive protein | Breast Cancer Specific Survival | AJCC Stage, Tumor Grade, IHC Subtype, Hormonal Therapy, Chemotherapy |
| C_reactive protein | Recurrence-free Survival | Race/Ethnicity, AJCC Stage, Tumor Grade, IHC Subtype, Hormonal Therapy, Chemotherapy, Radiation, Surgery |
| C_reactive protein | Disease-free Survival | Race/Ethnicity, AJCC Stage, Tumor Grade, IHC Subtype, Hormonal Therapy, Chemotherapy, Surgery |
| C_reactive protein | Invasive Disease-free Survival | Race/Ethnicity, AJCC Stage, Tumor Grade, IHC Subtype, Hormonal Therapy, Chemotherapy, Surgery |
| C_reactive protein | Second Primary Cancer-free Survival | Tumor Grade, IHC Subtype, Hormonal Therapy, Chemotherapy |
| C_reactive protein | Breast Event-free Survival | AJCC Stage, Tumor Grade, IHC Subtype, Hormonal Therapy |
| Calcium | Overall Survival | AJCC Stage, Tumor Grade, IHC Subtype, Hormonal Therapy, Chemotherapy, Radiation |
| Calcium | Breast Cancer Specific Survival | AJCC Stage, Tumor Grade, IHC Subtype, Hormonal Therapy, Chemotherapy |
| Calcium | Recurrence-free Survival | Race/Ethnicity, AJCC Stage, Tumor Grade, IHC Subtype, Hormonal Therapy, Chemotherapy, Radiation, Surgery |
| Calcium | Disease-free Survival | Race/Ethnicity, AJCC Stage, Tumor Grade, IHC Subtype, Hormonal Therapy, Chemotherapy, Surgery |
| Calcium | Invasive Disease-free Survival | Race/Ethnicity, AJCC Stage, Tumor Grade, IHC Subtype, Hormonal Therapy, Chemotherapy, Surgery |
| Calcium | Second Primary Cancer-free Survival | Tumor Grade, IHC Subtype, Hormonal Therapy, Chemotherapy |
| Calcium | Breast Event-free Survival | AJCC Stage, Tumor Grade, IHC Subtype, Hormonal Therapy |
| Total cholesterol | Overall Survival | AJCC Stage, Tumor Grade, IHC Subtype, Hormonal Therapy, Chemotherapy, Radiation |
| Total cholesterol | Breast Cancer Specific Survival | AJCC Stage, Tumor Grade, IHC Subtype, Hormonal Therapy, Chemotherapy |
| Total cholesterol | Recurrence-free Survival | Race/Ethnicity, AJCC Stage, Tumor Grade, IHC Subtype, Hormonal Therapy, Chemotherapy, Radiation, Surgery |
| Total cholesterol | Disease-free Survival | Race/Ethnicity, AJCC Stage, Tumor Grade, IHC Subtype, Hormonal Therapy, Chemotherapy, Surgery |
| Total cholesterol | Invasive Disease-free Survival | Race/Ethnicity, AJCC Stage, Tumor Grade, IHC Subtype, Hormonal Therapy, Chemotherapy, Surgery |
| Total cholesterol | Second Primary Cancer-free Survival | Tumor Grade, IHC Subtype, Hormonal Therapy, Chemotherapy |
| Total cholesterol | Breast Event-free Survival | AJCC Stage, Tumor Grade, IHC Subtype, Hormonal Therapy |
| Creatinine | Overall Survival | AJCC Stage, Tumor Grade, IHC Subtype, Hormonal Therapy, Chemotherapy, Radiation, Surgery |
| Creatinine | Breast Cancer Specific Survival | AJCC Stage, Tumor Grade, IHC Subtype, Hormonal Therapy, Chemotherapy |
| Creatinine | Recurrence-free Survival | Race/Ethnicity, AJCC Stage, Tumor Grade, IHC Subtype, Hormonal Therapy, Chemotherapy, Radiation, Surgery |
| Creatinine | Disease-free Survival | Race/Ethnicity, AJCC Stage, Tumor Grade, IHC Subtype, Hormonal Therapy, Chemotherapy, Surgery |
| Creatinine | Invasive Disease-free Survival | Race/Ethnicity, AJCC Stage, Tumor Grade, IHC Subtype, Hormonal Therapy, Chemotherapy, Surgery |
| Creatinine | Second Primary Cancer-free Survival | Tumor Grade, IHC Subtype, Hormonal Therapy, Chemotherapy |
| Creatinine | Breast Event-free Survival | AJCC Stage, Tumor Grade, IHC Subtype, Hormonal Therapy |
| Creatinine in urine | Overall Survival | AJCC Stage, Tumor Grade, IHC Subtype, Hormonal Therapy, Chemotherapy, Radiation |
| Creatinine in urine | Breast Cancer Specific Survival | AJCC Stage, Tumor Grade, IHC Subtype, Hormonal Therapy, Chemotherapy |
| Creatinine in urine | Recurrence-free Survival | Race/Ethnicity, AJCC Stage, Tumor Grade, IHC Subtype, Hormonal Therapy, Chemotherapy, Radiation, Surgery |
| Creatinine in urine | Disease-free Survival | Race/Ethnicity, AJCC Stage, Tumor Grade, IHC Subtype, Hormonal Therapy, Chemotherapy, Surgery |
| Creatinine in urine | Invasive Disease-free Survival | Race/Ethnicity, AJCC Stage, Tumor Grade, IHC Subtype, Hormonal Therapy, Chemotherapy, Surgery |
| Creatinine in urine | Second Primary Cancer-free Survival | Tumor Grade, IHC Subtype, Hormonal Therapy, Chemotherapy |
| Creatinine in urine | Breast Event-free Survival | AJCC Stage, Tumor Grade, IHC Subtype, Hormonal Therapy |
| Cystatin C | Overall Survival | AJCC Stage, Tumor Grade, IHC Subtype, Hormonal Therapy, Chemotherapy, Radiation |
| Cystatin C | Breast Cancer Specific Survival | AJCC Stage, Tumor Grade, IHC Subtype, Hormonal Therapy, Chemotherapy |
| Cystatin C | Recurrence-free Survival | Race/Ethnicity, AJCC Stage, Tumor Grade, IHC Subtype, Hormonal Therapy, Chemotherapy, Radiation, Surgery |
| Cystatin C | Disease-free Survival | Race/Ethnicity, AJCC Stage, Tumor Grade, IHC Subtype, Hormonal Therapy, Chemotherapy, Surgery |
| Cystatin C | Invasive Disease-free Survival | Race/Ethnicity, AJCC Stage, Tumor Grade, IHC Subtype, Hormonal Therapy, Chemotherapy, Surgery |
| Cystatin C | Second Primary Cancer-free Survival | Tumor Grade, IHC Subtype, Hormonal Therapy, Chemotherapy |
| Cystatin C | Breast Event-free Survival | AJCC Stage, Tumor Grade, IHC Subtype, Hormonal Therapy |
| Direct bilirubin | Overall Survival | AJCC Stage, Tumor Grade, IHC Subtype, Hormonal Therapy, Chemotherapy, Radiation |
| Direct bilirubin | Breast Cancer Specific Survival | AJCC Stage, Tumor Grade, IHC Subtype, Hormonal Therapy, Chemotherapy |
| Direct bilirubin | Recurrence-free Survival | Race/Ethnicity, AJCC Stage, Tumor Grade, IHC Subtype, Hormonal Therapy, Chemotherapy, Radiation, Surgery |
| Direct bilirubin | Disease-free Survival | Race/Ethnicity, AJCC Stage, Tumor Grade, IHC Subtype, Hormonal Therapy, Chemotherapy, Surgery |
| Direct bilirubin | Invasive Disease-free Survival | Race/Ethnicity, AJCC Stage, Tumor Grade, IHC Subtype, Hormonal Therapy, Chemotherapy, Surgery |
| Direct bilirubin | Second Primary Cancer-free Survival | Tumor Grade, IHC Subtype, Hormonal Therapy, Chemotherapy |
| Direct bilirubin | Breast Event-free Survival | AJCC Stage, Tumor Grade, IHC Subtype, Hormonal Therapy |
| eGFR | Overall Survival | AJCC Stage, Tumor Grade, IHC Subtype, Hormonal Therapy, Chemotherapy, Radiation, Surgery |
| eGFR | Breast Cancer Specific Survival | AJCC Stage, Tumor Grade, IHC Subtype, Hormonal Therapy, Chemotherapy |
| eGFR | Recurrence-free Survival | Race/Ethnicity, AJCC Stage, Tumor Grade, IHC Subtype, Hormonal Therapy, Chemotherapy, Radiation, Surgery |
| eGFR | Disease-free Survival | Race/Ethnicity, AJCC Stage, Tumor Grade, IHC Subtype, Hormonal Therapy, Chemotherapy, Surgery |
| eGFR | Invasive Disease-free Survival | Race/Ethnicity, AJCC Stage, Tumor Grade, IHC Subtype, Hormonal Therapy, Chemotherapy, Surgery |
| eGFR | Second Primary Cancer-free Survival | Tumor Grade, IHC Subtype, Hormonal Therapy, Chemotherapy |
| eGFR | Breast Event-free Survival | AJCC Stage, Tumor Grade, IHC Subtype, Hormonal Therapy |
| Gamma glutamyltransferase | Overall Survival | AJCC Stage, Tumor Grade, IHC Subtype, Hormonal Therapy, Chemotherapy, Radiation |
| Gamma glutamyltransferase | Breast Cancer Specific Survival | AJCC Stage, Tumor Grade, IHC Subtype, Hormonal Therapy, Chemotherapy |
| Gamma glutamyltransferase | Recurrence-free Survival | Race/Ethnicity, AJCC Stage, Tumor Grade, IHC Subtype, Hormonal Therapy, Chemotherapy, Radiation, Surgery |
| Gamma glutamyltransferase | Disease-free Survival | Race/Ethnicity, AJCC Stage, Tumor Grade, IHC Subtype, Hormonal Therapy, Chemotherapy, Surgery |
| Gamma glutamyltransferase | Invasive Disease-free Survival | Race/Ethnicity, AJCC Stage, Tumor Grade, IHC Subtype, Hormonal Therapy, Chemotherapy, Surgery |
| Gamma glutamyltransferase | Second Primary Cancer-free Survival | Tumor Grade, IHC Subtype, Hormonal Therapy, Chemotherapy |
| Gamma glutamyltransferase | Breast Event-free Survival | AJCC Stage, Tumor Grade, IHC Subtype, Hormonal Therapy |
| Non fasting glucose | Overall Survival | AJCC Stage, Tumor Grade, IHC Subtype, Hormonal Therapy, Chemotherapy, Radiation |
| Non fasting glucose | Breast Cancer Specific Survival | AJCC Stage, Tumor Grade, IHC Subtype, Hormonal Therapy, Chemotherapy |
| Non fasting glucose | Recurrence-free Survival | Race/Ethnicity, AJCC Stage, Tumor Grade, IHC Subtype, Hormonal Therapy, Chemotherapy, Radiation, Surgery |
| Non fasting glucose | Disease-free Survival | Race/Ethnicity, AJCC Stage, Tumor Grade, IHC Subtype, Hormonal Therapy, Chemotherapy, Surgery |
| Non fasting glucose | Invasive Disease-free Survival | Race/Ethnicity, AJCC Stage, Tumor Grade, IHC Subtype, Hormonal Therapy, Chemotherapy, Surgery |
| Non fasting glucose | Second Primary Cancer-free Survival | Tumor Grade, IHC Subtype, Hormonal Therapy, Chemotherapy |
| Non fasting glucose | Breast Event-free Survival | AJCC Stage, Tumor Grade, IHC Subtype, Hormonal Therapy |
| HbA1c | Overall Survival | AJCC Stage, Tumor Grade, IHC Subtype, Hormonal Therapy, Chemotherapy, Radiation |
| HbA1c | Breast Cancer Specific Survival | AJCC Stage, Tumor Grade, IHC Subtype, Hormonal Therapy, Chemotherapy |
| HbA1c | Recurrence-free Survival | Race/Ethnicity, AJCC Stage, Tumor Grade, IHC Subtype, Hormonal Therapy, Chemotherapy, Radiation, Surgery |
| HbA1c | Disease-free Survival | Race/Ethnicity, AJCC Stage, Tumor Grade, IHC Subtype, Hormonal Therapy, Chemotherapy, Surgery |
| HbA1c | Invasive Disease-free Survival | Race/Ethnicity, AJCC Stage, Tumor Grade, IHC Subtype, Hormonal Therapy, Chemotherapy, Surgery |
| HbA1c | Second Primary Cancer-free Survival | Tumor Grade, IHC Subtype, Hormonal Therapy, Chemotherapy |
| HbA1c | Breast Event-free Survival | AJCC Stage, Tumor Grade, IHC Subtype, Hormonal Therapy |
| HDL cholesterol | Overall Survival | AJCC Stage, Tumor Grade, IHC Subtype, Hormonal Therapy, Chemotherapy, Radiation, Surgery |
| HDL cholesterol | Breast Cancer Specific Survival | AJCC Stage, Tumor Grade, IHC Subtype, Hormonal Therapy, Chemotherapy |
| HDL cholesterol | Recurrence-free Survival | Race/Ethnicity, AJCC Stage, Tumor Grade, IHC Subtype, Hormonal Therapy, Chemotherapy, Radiation, Surgery |
| HDL cholesterol | Disease-free Survival | Race/Ethnicity, AJCC Stage, Tumor Grade, IHC Subtype, Hormonal Therapy, Chemotherapy, Surgery |
| HDL cholesterol | Invasive Disease-free Survival | Race/Ethnicity, AJCC Stage, Tumor Grade, IHC Subtype, Hormonal Therapy, Chemotherapy, Surgery |
| HDL cholesterol | Second Primary Cancer-free Survival | Tumor Grade, IHC Subtype, Hormonal Therapy, Chemotherapy |
| HDL cholesterol | Breast Event-free Survival | AJCC Stage, Tumor Grade, IHC Subtype, Hormonal Therapy |
| IGF 1 | Overall Survival | AJCC Stage, Tumor Grade, IHC Subtype, Hormonal Therapy, Chemotherapy, Radiation |
| IGF 1 | Breast Cancer Specific Survival | AJCC Stage, Tumor Grade, IHC Subtype, Hormonal Therapy, Chemotherapy |
| IGF 1 | Recurrence-free Survival | Race/Ethnicity, AJCC Stage, Tumor Grade, IHC Subtype, Hormonal Therapy, Chemotherapy, Radiation, Surgery |
| IGF 1 | Disease-free Survival | Race/Ethnicity, AJCC Stage, Tumor Grade, IHC Subtype, Hormonal Therapy, Chemotherapy, Surgery |
| IGF 1 | Invasive Disease-free Survival | Race/Ethnicity, AJCC Stage, Tumor Grade, IHC Subtype, Hormonal Therapy, Chemotherapy, Surgery |
| IGF 1 | Second Primary Cancer-free Survival | Tumor Grade, IHC Subtype, Hormonal Therapy, Chemotherapy |
| IGF 1 | Breast Event-free Survival | AJCC Stage, Tumor Grade, IHC Subtype, Hormonal Therapy |
| LDL cholesterol | Overall Survival | AJCC Stage, Tumor Grade, IHC Subtype, Hormonal Therapy, Chemotherapy, Radiation, Surgery |
| LDL cholesterol | Breast Cancer Specific Survival | AJCC Stage, Tumor Grade, IHC Subtype, Hormonal Therapy, Chemotherapy |
| LDL cholesterol | Recurrence-free Survival | Race/Ethnicity, AJCC Stage, Tumor Grade, IHC Subtype, Hormonal Therapy, Chemotherapy, Radiation, Surgery |
| LDL cholesterol | Disease-free Survival | Race/Ethnicity, AJCC Stage, Tumor Grade, IHC Subtype, Hormonal Therapy, Chemotherapy, Surgery |
| LDL cholesterol | Invasive Disease-free Survival | Race/Ethnicity, AJCC Stage, Tumor Grade, IHC Subtype, Hormonal Therapy, Chemotherapy, Surgery |
| LDL cholesterol | Second Primary Cancer-free Survival | Tumor Grade, IHC Subtype, Hormonal Therapy, Chemotherapy |
| LDL cholesterol | Breast Event-free Survival | AJCC Stage, Tumor Grade, IHC Subtype, Hormonal Therapy |
| Lipoprotein A | Overall Survival | AJCC Stage, Tumor Grade, IHC Subtype, Hormonal Therapy, Chemotherapy, Radiation |
| Lipoprotein A | Breast Cancer Specific Survival | AJCC Stage, Tumor Grade, IHC Subtype, Hormonal Therapy, Chemotherapy |
| Lipoprotein A | Recurrence-free Survival | Race/Ethnicity, AJCC Stage, Tumor Grade, IHC Subtype, Hormonal Therapy, Chemotherapy, Radiation, Surgery |
| Lipoprotein A | Disease-free Survival | Race/Ethnicity, AJCC Stage, Tumor Grade, IHC Subtype, Hormonal Therapy, Chemotherapy, Surgery |
| Lipoprotein A | Invasive Disease-free Survival | Race/Ethnicity, AJCC Stage, Tumor Grade, IHC Subtype, Hormonal Therapy, Chemotherapy, Surgery |
| Lipoprotein A | Second Primary Cancer-free Survival | Tumor Grade, IHC Subtype, Hormonal Therapy, Chemotherapy |
| Lipoprotein A | Breast Event-free Survival | AJCC Stage, Tumor Grade, IHC Subtype, Hormonal Therapy |
| Microalbumin in urine | Overall Survival | AJCC Stage, Tumor Grade, IHC Subtype, Hormonal Therapy, Chemotherapy, Radiation |
| Microalbumin in urine | Breast Cancer Specific Survival | AJCC Stage, Tumor Grade, IHC Subtype, Hormonal Therapy, Chemotherapy |
| Microalbumin in urine | Recurrence-free Survival | Race/Ethnicity, AJCC Stage, Tumor Grade, IHC Subtype, Hormonal Therapy, Chemotherapy, Radiation, Surgery |
| Microalbumin in urine | Disease-free Survival | Race/Ethnicity, AJCC Stage, Tumor Grade, IHC Subtype, Hormonal Therapy, Chemotherapy, Surgery |
| Microalbumin in urine | Invasive Disease-free Survival | Race/Ethnicity, AJCC Stage, Tumor Grade, IHC Subtype, Hormonal Therapy, Chemotherapy, Surgery |
| Microalbumin in urine | Second Primary Cancer-free Survival | Tumor Grade, IHC Subtype, Hormonal Therapy, Chemotherapy |
| Microalbumin in urine | Breast Event-free Survival | AJCC Stage, Tumor Grade, IHC Subtype, Hormonal Therapy |
| Non albumin protein | Overall Survival | AJCC Stage, Tumor Grade, IHC Subtype, Hormonal Therapy, Chemotherapy, Radiation, Surgery |
| Non albumin protein | Breast Cancer Specific Survival | AJCC Stage, Tumor Grade, IHC Subtype, Hormonal Therapy, Chemotherapy |
| Non albumin protein | Recurrence-free Survival | Race/Ethnicity, AJCC Stage, Tumor Grade, IHC Subtype, Hormonal Therapy, Chemotherapy, Radiation, Surgery |
| Non albumin protein | Disease-free Survival | Race/Ethnicity, AJCC Stage, Tumor Grade, IHC Subtype, Hormonal Therapy, Chemotherapy, Surgery |
| Non albumin protein | Invasive Disease-free Survival | Race/Ethnicity, AJCC Stage, Tumor Grade, IHC Subtype, Hormonal Therapy, Chemotherapy, Surgery |
| Non albumin protein | Second Primary Cancer-free Survival | Tumor Grade, IHC Subtype, Hormonal Therapy, Chemotherapy |
| Non albumin protein | Breast Event-free Survival | AJCC Stage, Tumor Grade, IHC Subtype, Hormonal Therapy |
| Phosphate | Overall Survival | AJCC Stage, Tumor Grade, IHC Subtype, Hormonal Therapy, Chemotherapy, Radiation, Surgery |
| Phosphate | Breast Cancer Specific Survival | AJCC Stage, Tumor Grade, IHC Subtype, Hormonal Therapy, Chemotherapy |
| Phosphate | Recurrence-free Survival | Race/Ethnicity, AJCC Stage, Tumor Grade, IHC Subtype, Hormonal Therapy, Chemotherapy, Radiation, Surgery |
| Phosphate | Disease-free Survival | Race/Ethnicity, AJCC Stage, Tumor Grade, IHC Subtype, Hormonal Therapy, Chemotherapy, Surgery |
| Phosphate | Invasive Disease-free Survival | Race/Ethnicity, AJCC Stage, Tumor Grade, IHC Subtype, Hormonal Therapy, Chemotherapy, Surgery |
| Phosphate | Second Primary Cancer-free Survival | Tumor Grade, IHC Subtype, Hormonal Therapy, Chemotherapy |
| Phosphate | Breast Event-free Survival | AJCC Stage, Tumor Grade, IHC Subtype, Hormonal Therapy |
| Potassium in urine | Overall Survival | AJCC Stage, Tumor Grade, IHC Subtype, Hormonal Therapy, Chemotherapy, Radiation |
| Potassium in urine | Breast Cancer Specific Survival | AJCC Stage, Tumor Grade, IHC Subtype, Hormonal Therapy, Chemotherapy |
| Potassium in urine | Recurrence-free Survival | Race/Ethnicity, AJCC Stage, Tumor Grade, IHC Subtype, Hormonal Therapy, Chemotherapy, Radiation, Surgery |
| Potassium in urine | Disease-free Survival | Race/Ethnicity, AJCC Stage, Tumor Grade, IHC Subtype, Hormonal Therapy, Chemotherapy, Surgery |
| Potassium in urine | Invasive Disease-free Survival | Race/Ethnicity, AJCC Stage, Tumor Grade, IHC Subtype, Hormonal Therapy, Chemotherapy, Surgery |
| Potassium in urine | Second Primary Cancer-free Survival | Tumor Grade, IHC Subtype, Hormonal Therapy, Chemotherapy |
| Potassium in urine | Breast Event-free Survival | AJCC Stage, Tumor Grade, IHC Subtype, Hormonal Therapy |
| SHBG | Overall Survival | AJCC Stage, Tumor Grade, IHC Subtype, Hormonal Therapy, Chemotherapy, Radiation |
| SHBG | Breast Cancer Specific Survival | AJCC Stage, Tumor Grade, IHC Subtype, Hormonal Therapy, Chemotherapy |
| SHBG | Recurrence-free Survival | Race/Ethnicity, AJCC Stage, Tumor Grade, IHC Subtype, Hormonal Therapy, Chemotherapy, Radiation, Surgery |
| SHBG | Disease-free Survival | Race/Ethnicity, AJCC Stage, Tumor Grade, IHC Subtype, Hormonal Therapy, Chemotherapy, Surgery |
| SHBG | Invasive Disease-free Survival | Race/Ethnicity, AJCC Stage, Tumor Grade, IHC Subtype, Hormonal Therapy, Chemotherapy, Surgery |
| SHBG | Second Primary Cancer-free Survival | Tumor Grade, IHC Subtype, Hormonal Therapy, Chemotherapy |
| SHBG | Breast Event-free Survival | AJCC Stage, Tumor Grade, IHC Subtype, Hormonal Therapy |
| Sodium in urine | Overall Survival | AJCC Stage, Tumor Grade, IHC Subtype, Hormonal Therapy, Chemotherapy, Radiation |
| Sodium in urine | Breast Cancer Specific Survival | AJCC Stage, Tumor Grade, IHC Subtype, Hormonal Therapy, Chemotherapy |
| Sodium in urine | Recurrence-free Survival | Race/Ethnicity, AJCC Stage, Tumor Grade, IHC Subtype, Hormonal Therapy, Chemotherapy, Radiation, Surgery |
| Sodium in urine | Disease-free Survival | Race/Ethnicity, AJCC Stage, Tumor Grade, IHC Subtype, Hormonal Therapy, Chemotherapy, Surgery |
| Sodium in urine | Invasive Disease-free Survival | Race/Ethnicity, AJCC Stage, Tumor Grade, IHC Subtype, Hormonal Therapy, Chemotherapy, Surgery |
| Sodium in urine | Second Primary Cancer-free Survival | Tumor Grade, IHC Subtype, Hormonal Therapy, Chemotherapy |
| Sodium in urine | Breast Event-free Survival | AJCC Stage, Tumor Grade, IHC Subtype, Hormonal Therapy |
| Total bilirubin | Overall Survival | AJCC Stage, Tumor Grade, IHC Subtype, Hormonal Therapy, Chemotherapy, Radiation |
| Total bilirubin | Breast Cancer Specific Survival | AJCC Stage, Tumor Grade, IHC Subtype, Hormonal Therapy, Chemotherapy |
| Total bilirubin | Recurrence-free Survival | Race/Ethnicity, AJCC Stage, Tumor Grade, IHC Subtype, Hormonal Therapy, Chemotherapy, Radiation, Surgery |
| Total bilirubin | Disease-free Survival | Race/Ethnicity, AJCC Stage, Tumor Grade, IHC Subtype, Hormonal Therapy, Chemotherapy, Surgery |
| Total bilirubin | Invasive Disease-free Survival | Race/Ethnicity, AJCC Stage, Tumor Grade, IHC Subtype, Hormonal Therapy, Chemotherapy, Surgery |
| Total bilirubin | Second Primary Cancer-free Survival | Tumor Grade, IHC Subtype, Hormonal Therapy, Chemotherapy |
| Total bilirubin | Breast Event-free Survival | AJCC Stage, Tumor Grade, IHC Subtype, Hormonal Therapy |
| Total protein | Overall Survival | AJCC Stage, Tumor Grade, IHC Subtype, Hormonal Therapy, Chemotherapy, Radiation, Surgery |
| Total protein | Breast Cancer Specific Survival | AJCC Stage, Tumor Grade, IHC Subtype, Hormonal Therapy, Chemotherapy |
| Total protein | Recurrence-free Survival | Race/Ethnicity, AJCC Stage, Tumor Grade, IHC Subtype, Hormonal Therapy, Chemotherapy, Radiation, Surgery |
| Total protein | Disease-free Survival | Race/Ethnicity, AJCC Stage, Tumor Grade, IHC Subtype, Hormonal Therapy, Chemotherapy, Surgery |
| Total protein | Invasive Disease-free Survival | Race/Ethnicity, AJCC Stage, Tumor Grade, IHC Subtype, Hormonal Therapy, Chemotherapy, Surgery |
| Total protein | Second Primary Cancer-free Survival | Tumor Grade, IHC Subtype, Hormonal Therapy, Chemotherapy |
| Total protein | Breast Event-free Survival | AJCC Stage, Tumor Grade, IHC Subtype, Hormonal Therapy |
| Triglyceride | Overall Survival | AJCC Stage, Tumor Grade, IHC Subtype, Hormonal Therapy, Chemotherapy, Radiation |
| Triglyceride | Breast Cancer Specific Survival | AJCC Stage, Tumor Grade, IHC Subtype, Hormonal Therapy, Chemotherapy |
| Triglyceride | Recurrence-free Survival | Race/Ethnicity, AJCC Stage, Tumor Grade, IHC Subtype, Hormonal Therapy, Chemotherapy, Radiation, Surgery |
| Triglyceride | Disease-free Survival | Race/Ethnicity, AJCC Stage, Tumor Grade, IHC Subtype, Hormonal Therapy, Chemotherapy, Surgery |
| Triglyceride | Invasive Disease-free Survival | Race/Ethnicity, AJCC Stage, Tumor Grade, IHC Subtype, Hormonal Therapy, Chemotherapy, Surgery |
| Triglyceride | Second Primary Cancer-free Survival | Tumor Grade, IHC Subtype, Hormonal Therapy, Chemotherapy |
| Triglyceride | Breast Event-free Survival | AJCC Stage, Tumor Grade, IHC Subtype, Hormonal Therapy |
| Urate | Overall Survival | AJCC Stage, Tumor Grade, IHC Subtype, Hormonal Therapy, Chemotherapy, Radiation |
| Urate | Breast Cancer Specific Survival | AJCC Stage, Tumor Grade, IHC Subtype, Hormonal Therapy, Chemotherapy |
| Urate | Recurrence-free Survival | Race/Ethnicity, AJCC Stage, Tumor Grade, IHC Subtype, Hormonal Therapy, Chemotherapy, Radiation, Surgery |
| Urate | Disease-free Survival | Race/Ethnicity, AJCC Stage, Tumor Grade, IHC Subtype, Hormonal Therapy, Chemotherapy, Surgery |
| Urate | Invasive Disease-free Survival | Race/Ethnicity, AJCC Stage, Tumor Grade, IHC Subtype, Hormonal Therapy, Chemotherapy, Surgery |
| Urate | Second Primary Cancer-free Survival | Tumor Grade, IHC Subtype, Hormonal Therapy, Chemotherapy |
| Urate | Breast Event-free Survival | AJCC Stage, Tumor Grade, IHC Subtype, Hormonal Therapy |
| Urea serum | Overall Survival | AJCC Stage, Tumor Grade, IHC Subtype, Hormonal Therapy, Chemotherapy, Radiation |
| Urea serum | Breast Cancer Specific Survival | AJCC Stage, Tumor Grade, IHC Subtype, Hormonal Therapy, Chemotherapy |
| Urea serum | Recurrence-free Survival | Race/Ethnicity, AJCC Stage, Tumor Grade, IHC Subtype, Hormonal Therapy, Chemotherapy, Radiation, Surgery |
| Urea serum | Disease-free Survival | Race/Ethnicity, AJCC Stage, Tumor Grade, IHC Subtype, Hormonal Therapy, Chemotherapy, Surgery |
| Urea serum | Invasive Disease-free Survival | Race/Ethnicity, AJCC Stage, Tumor Grade, IHC Subtype, Hormonal Therapy, Chemotherapy, Surgery |
| Urea serum | Second Primary Cancer-free Survival | Tumor Grade, IHC Subtype, Hormonal Therapy, Chemotherapy |
| Urea serum | Breast Event-free Survival | AJCC Stage, Tumor Grade, IHC Subtype, Hormonal Therapy |
| Angina | Overall Survival | AJCC Stage, Tumor Grade, IHC Subtype, Hormonal Therapy, Chemotherapy, Radiation |
| Angina | Breast Cancer Specific Survival | AJCC Stage, Tumor Grade, IHC Subtype, Hormonal Therapy, Chemotherapy |
| Angina | Recurrence-free Survival | Race/Ethnicity, AJCC Stage, Tumor Grade, IHC Subtype, Hormonal Therapy, Chemotherapy, Radiation, Surgery |
| Angina | Disease-free Survival | Race/Ethnicity, AJCC Stage, Tumor Grade, IHC Subtype, Hormonal Therapy, Chemotherapy, Surgery |
| Angina | Invasive Disease-free Survival | Race/Ethnicity, AJCC Stage, Tumor Grade, IHC Subtype, Hormonal Therapy, Chemotherapy, Surgery |
| Angina | Second Primary Cancer-free Survival | Tumor Grade, IHC Subtype, Hormonal Therapy, Chemotherapy |
| Angina | Breast Event-free Survival | AJCC Stage, Tumor Grade, IHC Subtype, Hormonal Therapy |
| Hypertension | Overall Survival | AJCC Stage, Tumor Grade, IHC Subtype, Hormonal Therapy, Chemotherapy, Radiation |
| Hypertension | Breast Cancer Specific Survival | AJCC Stage, Tumor Grade, IHC Subtype, Hormonal Therapy, Chemotherapy |
| Hypertension | Recurrence-free Survival | Race/Ethnicity, AJCC Stage, Tumor Grade, IHC Subtype, Hormonal Therapy, Chemotherapy, Radiation, Surgery |
| Hypertension | Disease-free Survival | Race/Ethnicity, AJCC Stage, Tumor Grade, IHC Subtype, Hormonal Therapy, Chemotherapy, Surgery |
| Hypertension | Invasive Disease-free Survival | Race/Ethnicity, AJCC Stage, Tumor Grade, IHC Subtype, Hormonal Therapy, Chemotherapy, Surgery |
| Hypertension | Second Primary Cancer-free Survival | Tumor Grade, IHC Subtype, Hormonal Therapy, Chemotherapy |
| Hypertension | Breast Event-free Survival | AJCC Stage, Tumor Grade, IHC Subtype, Hormonal Therapy |
| Kidney failure | Overall Survival | AJCC Stage, Tumor Grade, IHC Subtype, Hormonal Therapy, Chemotherapy, Radiation |
| Kidney failure | Breast Cancer Specific Survival | AJCC Stage, Tumor Grade, IHC Subtype, Hormonal Therapy, Chemotherapy |
| Kidney failure | Recurrence-free Survival | Race/Ethnicity, AJCC Stage, Tumor Grade, IHC Subtype, Hormonal Therapy, Chemotherapy, Radiation, Surgery |
| Kidney failure | Disease-free Survival | Race/Ethnicity, AJCC Stage, Tumor Grade, IHC Subtype, Hormonal Therapy, Chemotherapy, Surgery |
| Kidney failure | Invasive Disease-free Survival | Race/Ethnicity, AJCC Stage, Tumor Grade, IHC Subtype, Hormonal Therapy, Chemotherapy, Surgery |
| Kidney failure | Second Primary Cancer-free Survival | Tumor Grade, IHC Subtype, Hormonal Therapy, Chemotherapy |
| Kidney failure | Breast Event-free Survival | AJCC Stage, Tumor Grade, IHC Subtype, Hormonal Therapy |
| Heart failure | Overall Survival | AJCC Stage, Tumor Grade, IHC Subtype, Hormonal Therapy, Chemotherapy, Radiation |
| Heart failure | Breast Cancer Specific Survival | AJCC Stage, Tumor Grade, IHC Subtype, Hormonal Therapy, Chemotherapy |
| Heart failure | Recurrence-free Survival | Race/Ethnicity, AJCC Stage, Tumor Grade, IHC Subtype, Hormonal Therapy, Chemotherapy, Radiation, Surgery |
| Heart failure | Disease-free Survival | Race/Ethnicity, AJCC Stage, Tumor Grade, IHC Subtype, Hormonal Therapy, Chemotherapy, Surgery |
| Heart failure | Invasive Disease-free Survival | Race/Ethnicity, AJCC Stage, Tumor Grade, IHC Subtype, Hormonal Therapy, Chemotherapy, Surgery |
| Heart failure | Second Primary Cancer-free Survival | Tumor Grade, IHC Subtype, Hormonal Therapy, Chemotherapy |
| Heart failure | Breast Event-free Survival | AJCC Stage, Tumor Grade, IHC Subtype, Hormonal Therapy |
| Myocardial infarction | Overall Survival | AJCC Stage, Tumor Grade, IHC Subtype, Hormonal Therapy, Chemotherapy, Radiation |
| Myocardial infarction | Breast Cancer Specific Survival | AJCC Stage, Tumor Grade, IHC Subtype, Hormonal Therapy, Chemotherapy |
| Myocardial infarction | Recurrence-free Survival | Race/Ethnicity, AJCC Stage, Tumor Grade, IHC Subtype, Hormonal Therapy, Chemotherapy, Radiation, Surgery |
| Myocardial infarction | Disease-free Survival | Race/Ethnicity, AJCC Stage, Tumor Grade, IHC Subtype, Hormonal Therapy, Chemotherapy, Surgery |
| Myocardial infarction | Invasive Disease-free Survival | Race/Ethnicity, AJCC Stage, Tumor Grade, IHC Subtype, Hormonal Therapy, Chemotherapy, Surgery |
| Myocardial infarction | Second Primary Cancer-free Survival | Tumor Grade, IHC Subtype, Hormonal Therapy, Chemotherapy |
| Myocardial infarction | Breast Event-free Survival | AJCC Stage, Tumor Grade, IHC Subtype, Hormonal Therapy |
| Body mass index early life | Overall Survival | AJCC Stage, Tumor Grade, IHC Subtype, Hormonal Therapy, Chemotherapy, Radiation |
| Body mass index early life | Breast Cancer Specific Survival | AJCC Stage, Tumor Grade, IHC Subtype, Hormonal Therapy, Chemotherapy |
| Body mass index early life | Recurrence-free Survival | Race/Ethnicity, AJCC Stage, Tumor Grade, IHC Subtype, Hormonal Therapy, Chemotherapy, Radiation, Surgery |
| Body mass index early life | Disease-free Survival | Race/Ethnicity, AJCC Stage, Tumor Grade, IHC Subtype, Hormonal Therapy, Chemotherapy, Surgery |
| Body mass index early life | Invasive Disease-free Survival | Race/Ethnicity, AJCC Stage, Tumor Grade, IHC Subtype, Hormonal Therapy, Chemotherapy, Surgery |
| Body mass index early life | Second Primary Cancer-free Survival | Tumor Grade, IHC Subtype, Hormonal Therapy, Chemotherapy |
| Body mass index early life | Breast Event-free Survival | AJCC Stage, Tumor Grade, IHC Subtype, Hormonal Therapy |
| Chronic kidney disease | Overall Survival | AJCC Stage, Tumor Grade, IHC Subtype, Hormonal Therapy, Chemotherapy, Radiation |
| Chronic kidney disease | Breast Cancer Specific Survival | AJCC Stage, Tumor Grade, IHC Subtype, Hormonal Therapy, Chemotherapy |
| Chronic kidney disease | Recurrence-free Survival | Race/Ethnicity, AJCC Stage, Tumor Grade, IHC Subtype, Hormonal Therapy, Chemotherapy, Radiation, Surgery |
| Chronic kidney disease | Disease-free Survival | Race/Ethnicity, AJCC Stage, Tumor Grade, IHC Subtype, Hormonal Therapy, Chemotherapy, Surgery |
| Chronic kidney disease | Invasive Disease-free Survival | Race/Ethnicity, AJCC Stage, Tumor Grade, IHC Subtype, Hormonal Therapy, Chemotherapy, Surgery |
| Chronic kidney disease | Second Primary Cancer-free Survival | Tumor Grade, IHC Subtype, Hormonal Therapy, Chemotherapy |
| Chronic kidney disease | Breast Event-free Survival | AJCC Stage, Tumor Grade, IHC Subtype, Hormonal Therapy |
| Urea urine | Overall Survival | AJCC Stage, Tumor Grade, IHC Subtype, Hormonal Therapy, Chemotherapy, Radiation |
| Urea urine | Breast Cancer Specific Survival | AJCC Stage, Tumor Grade, IHC Subtype, Hormonal Therapy, Chemotherapy |
| Urea urine | Recurrence-free Survival | Race/Ethnicity, AJCC Stage, Tumor Grade, IHC Subtype, Hormonal Therapy, Chemotherapy, Radiation, Surgery |
| Urea urine | Disease-free Survival | Race/Ethnicity, AJCC Stage, Tumor Grade, IHC Subtype, Hormonal Therapy, Chemotherapy, Surgery |
| Urea urine | Invasive Disease-free Survival | Race/Ethnicity, AJCC Stage, Tumor Grade, IHC Subtype, Hormonal Therapy, Chemotherapy, Surgery |
| Urea urine | Second Primary Cancer-free Survival | Tumor Grade, IHC Subtype, Hormonal Therapy, Chemotherapy |
| Urea urine | Breast Event-free Survival | AJCC Stage, Tumor Grade, IHC Subtype, Hormonal Therapy |
| Body fat percentage | Overall Survival | AJCC Stage, Tumor Grade, IHC Subtype, Hormonal Therapy, Chemotherapy, Radiation |
| Body fat percentage | Breast Cancer Specific Survival | AJCC Stage, Tumor Grade, IHC Subtype, Hormonal Therapy, Chemotherapy |
| Body fat percentage | Recurrence-free Survival | Race/Ethnicity, AJCC Stage, Tumor Grade, IHC Subtype, Hormonal Therapy, Chemotherapy, Radiation, Surgery |
| Body fat percentage | Disease-free Survival | Race/Ethnicity, AJCC Stage, Tumor Grade, IHC Subtype, Hormonal Therapy, Chemotherapy, Surgery |
| Body fat percentage | Invasive Disease-free Survival | Race/Ethnicity, AJCC Stage, Tumor Grade, IHC Subtype, Hormonal Therapy, Chemotherapy, Surgery |
| Body fat percentage | Second Primary Cancer-free Survival | Tumor Grade, IHC Subtype, Hormonal Therapy, Chemotherapy |
| Body fat percentage | Breast Event-free Survival | AJCC Stage, Tumor Grade, IHC Subtype, Hormonal Therapy |
| Diastolic blood pressure | Overall Survival | AJCC Stage, Tumor Grade, IHC Subtype, Hormonal Therapy, Chemotherapy, Radiation |
| Diastolic blood pressure | Breast Cancer Specific Survival | AJCC Stage, Tumor Grade, IHC Subtype, Hormonal Therapy, Chemotherapy |
| Diastolic blood pressure | Recurrence-free Survival | Race/Ethnicity, AJCC Stage, Tumor Grade, IHC Subtype, Hormonal Therapy, Chemotherapy, Radiation, Surgery |
| Diastolic blood pressure | Disease-free Survival | Race/Ethnicity, AJCC Stage, Tumor Grade, IHC Subtype, Hormonal Therapy, Chemotherapy, Surgery |
| Diastolic blood pressure | Invasive Disease-free Survival | Race/Ethnicity, AJCC Stage, Tumor Grade, IHC Subtype, Hormonal Therapy, Chemotherapy, Surgery |
| Diastolic blood pressure | Second Primary Cancer-free Survival | Tumor Grade, IHC Subtype, Hormonal Therapy, Chemotherapy |
| Diastolic blood pressure | Breast Event-free Survival | AJCC Stage, Tumor Grade, IHC Subtype, Hormonal Therapy |
| Systolic blood pressure | Overall Survival | AJCC Stage, Tumor Grade, IHC Subtype, Hormonal Therapy, Chemotherapy, Radiation |
| Systolic blood pressure | Breast Cancer Specific Survival | AJCC Stage, Tumor Grade, IHC Subtype, Hormonal Therapy, Chemotherapy |
| Systolic blood pressure | Recurrence-free Survival | Race/Ethnicity, AJCC Stage, Tumor Grade, IHC Subtype, Hormonal Therapy, Chemotherapy, Radiation, Surgery |
| Systolic blood pressure | Disease-free Survival | Race/Ethnicity, AJCC Stage, Tumor Grade, IHC Subtype, Hormonal Therapy, Chemotherapy, Surgery |
| Systolic blood pressure | Invasive Disease-free Survival | Race/Ethnicity, AJCC Stage, Tumor Grade, IHC Subtype, Hormonal Therapy, Chemotherapy, Surgery |
| Systolic blood pressure | Second Primary Cancer-free Survival | Tumor Grade, IHC Subtype, Hormonal Therapy, Chemotherapy |
| Systolic blood pressure | Breast Event-free Survival | AJCC Stage, Tumor Grade, IHC Subtype, Hormonal Therapy |
| Waist circumference | Overall Survival | AJCC Stage, Tumor Grade, IHC Subtype, Hormonal Therapy, Chemotherapy, Radiation |
| Waist circumference | Breast Cancer Specific Survival | AJCC Stage, Tumor Grade, IHC Subtype, Hormonal Therapy, Chemotherapy |
| Waist circumference | Recurrence-free Survival | Race/Ethnicity, AJCC Stage, Tumor Grade, IHC Subtype, Hormonal Therapy, Chemotherapy, Radiation, Surgery |
| Waist circumference | Disease-free Survival | Race/Ethnicity, AJCC Stage, Tumor Grade, IHC Subtype, Hormonal Therapy, Chemotherapy, Surgery |
| Waist circumference | Invasive Disease-free Survival | Race/Ethnicity, AJCC Stage, Tumor Grade, IHC Subtype, Hormonal Therapy, Chemotherapy, Surgery |
| Waist circumference | Second Primary Cancer-free Survival | Tumor Grade, IHC Subtype, Hormonal Therapy, Chemotherapy |
| Waist circumference | Breast Event-free Survival | AJCC Stage, Tumor Grade, IHC Subtype, Hormonal Therapy |
| body mass index adult | Overall Survival | AJCC Stage, Tumor Grade, IHC Subtype, Hormonal Therapy, Chemotherapy, Radiation |
| body mass index adult | Breast Cancer Specific Survival | AJCC Stage, Tumor Grade, IHC Subtype, Hormonal Therapy, Chemotherapy |
| body mass index adult | Recurrence-free Survival | Race/Ethnicity, AJCC Stage, Tumor Grade, IHC Subtype, Hormonal Therapy, Chemotherapy, Radiation, Surgery |
| body mass index adult | Disease-free Survival | Race/Ethnicity, AJCC Stage, Tumor Grade, IHC Subtype, Hormonal Therapy, Chemotherapy, Surgery |
| body mass index adult | Invasive Disease-free Survival | Race/Ethnicity, AJCC Stage, Tumor Grade, IHC Subtype, Hormonal Therapy, Chemotherapy, Surgery |
| body mass index adult | Second Primary Cancer-free Survival | Tumor Grade, IHC Subtype, Hormonal Therapy, Chemotherapy |
| body mass index adult | Breast Event-free Survival | AJCC Stage, Tumor Grade, IHC Subtype, Hormonal Therapy |
| Body weight | Overall Survival | AJCC Stage, Tumor Grade, IHC Subtype, Hormonal Therapy, Chemotherapy, Radiation |
| Body weight | Breast Cancer Specific Survival | AJCC Stage, Tumor Grade, IHC Subtype, Hormonal Therapy, Chemotherapy |
| Body weight | Recurrence-free Survival | Race/Ethnicity, AJCC Stage, Tumor Grade, IHC Subtype, Hormonal Therapy, Chemotherapy, Radiation, Surgery |
| Body weight | Disease-free Survival | Race/Ethnicity, AJCC Stage, Tumor Grade, IHC Subtype, Hormonal Therapy, Chemotherapy, Surgery |
| Body weight | Invasive Disease-free Survival | Race/Ethnicity, AJCC Stage, Tumor Grade, IHC Subtype, Hormonal Therapy, Chemotherapy, Surgery |
| Body weight | Second Primary Cancer-free Survival | Tumor Grade, IHC Subtype, Hormonal Therapy, Chemotherapy |
| Body weight | Breast Event-free Survival | AJCC Stage, Tumor Grade, IHC Subtype, Hormonal Therapy |

**Footnote:** A table that presents the results for PGS-survival outcome associations at $P<0.05$ in individuals that self-reported their race as "White." Each row represents a PGS tertile relationship with a given survival outcome. The columns include the following information: PGS Trait, tertile for the given trait, hazard ratio with 95% confidence intervals, P-value for the trait survival outcome association, survival outcome associated.
