# Supplementary material for: A Mendelian Randomization Analysis of 55 Genetically Predicted Metabolic Traits with Breast Cancer Survival Outcomes in the Pathways Study
Source: Cancer Res Commun. 2023 Jun 22;3(6):1104–12. doi: 10.1158/2767-9764.CRC-23-0047 (PMC10286812; doi:10.1158/2767-9764.CRC-23-0047)
Supplement: Supplementary Figure 1 — Dot plot of findings from analysis of 2,696 White individuals [file crc-23-0047-s04.docx]

#### **Supplemental Figure 1. Dot plot of findings from analysis of 2,696 White individuals.**


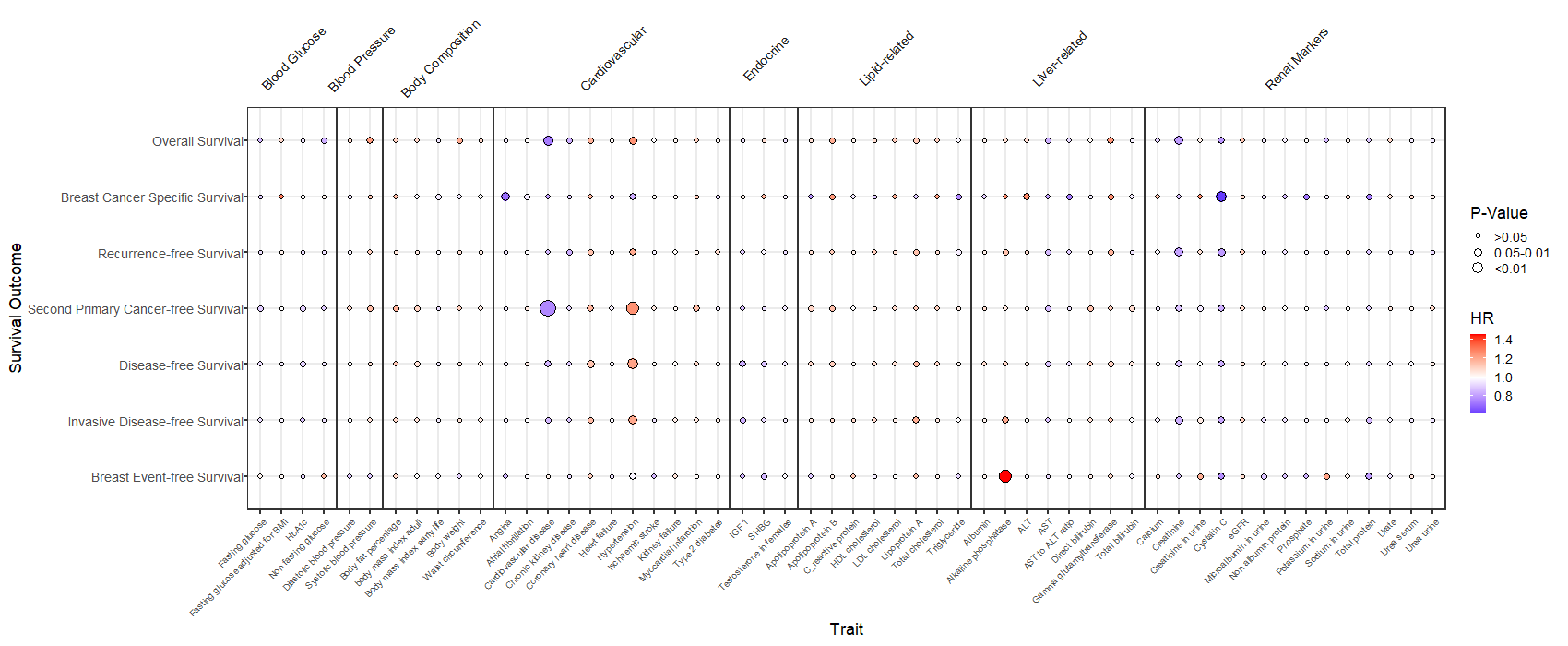


#### **Figure Legend.** A dot plot of associations in our analysis including only individuals who self-identified as "White." The x-axis represents the 55 PGS for metabolic traits and the y-axis represents the 7 survival outcomes. Each dot represents a PGS tertile (T3 vs. T1)-survival outcome association. The size of the point is inversely proportional to its P-value with a star denoting associations with $P<0.05$. The color of the point represents the hazard ratio. Bluer colors represent $HR<1.0$, and more red colors represent $HR>1.0$.
