# Supplementary material for: A Mendelian Randomization Analysis of 55 Genetically Predicted Metabolic Traits with Breast Cancer Survival Outcomes in the Pathways Study
Source: Cancer Res Commun. 2023 Jun 22;3(6):1104–12. doi: 10.1158/2767-9764.CRC-23-0047 (PMC10286812; doi:10.1158/2767-9764.CRC-23-0047)
Supplement: Supplementary Figure 2 — Dot plot of statistically-significant findings stratified by tumor estrogen receptor (ER) status in whole analytic population, with 3,260 ER+ and 640 ER- individuals [file crc-23-0047-s05.docx]

#### **Supplemental Figure 2. Dot plot of statistically-significant findings stratified by tumor estrogen receptor (ER) status in whole analytic population, with 3,260 ER+ and 640 ER- individuals.**

#### **
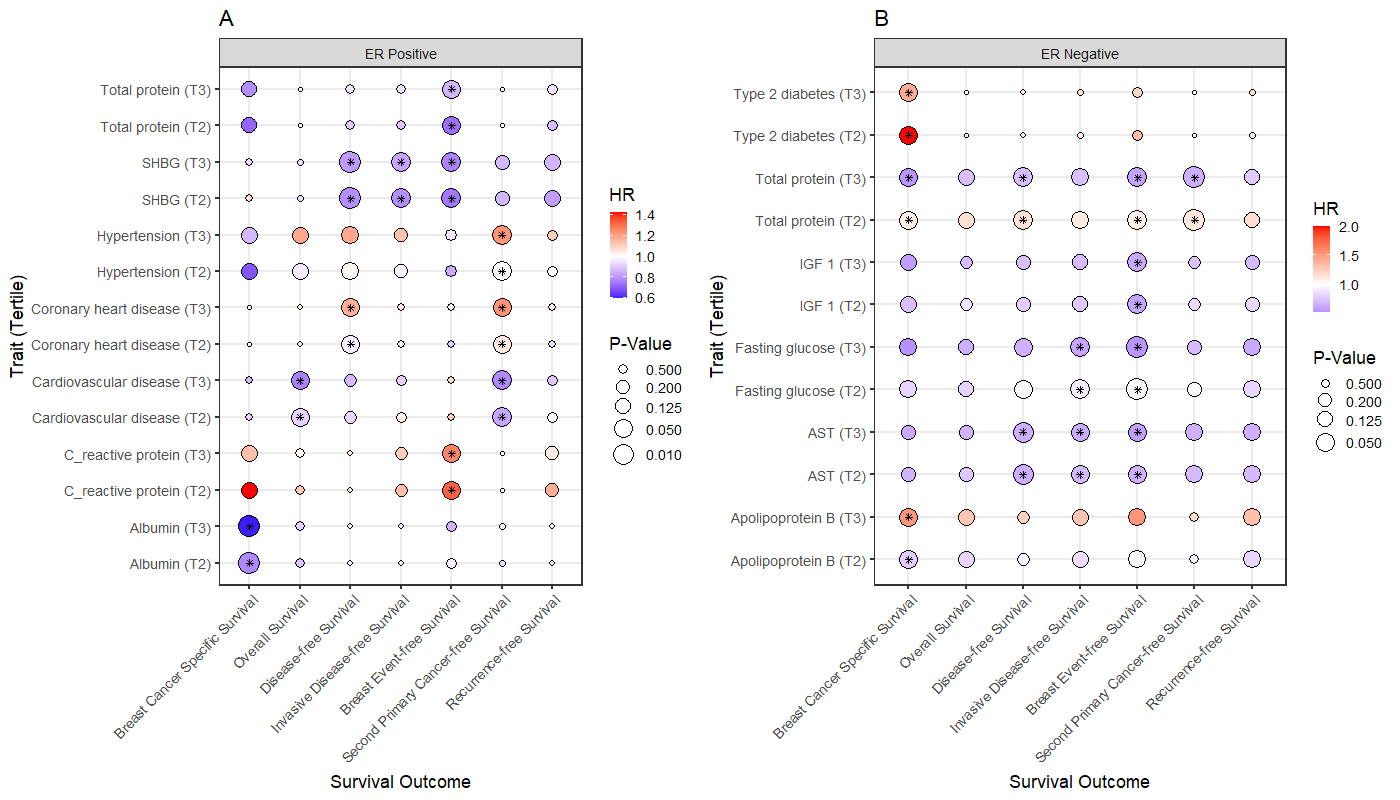
**

#### **Figure Legend:** Two dot plots for PGS-survival outcomes for ER+ (A) and ER- (B) individuals. The x-axis represents survival outcome, and the y-axis represents the trait for the respective PGS with its tertile in parentheses. The size of the point is inversely proportional to its P-value with a star denoting associations with $P<0.05$. The color of the point represents the hazard ratio. Bluer colors represent $HR<1.0$, and more red colors represent $HR>1.0$.
